# Sparse ensemble neural code for a complete vocal repertoire

**DOI:** 10.1016/j.celrep.2023.112034

**Published:** 2023-01-24

**Authors:** H. Robotka, L. Thomas, K. Yu, W. Wood, J.E. Elie, M. Gahr, F.E. Theunissen

**Affiliations:** 1 Max Planck Institute for Ornithology, Seewiesen, Germany; 2 University of California, Berkeley, Helen Wills Neuroscience Institute, Berkeley, CA, USA; 3 Department of Psychology and Integrative Biology, University of California, Berkeley, Berkeley, CA, USA; 4 Lead contact

## Abstract

The categorization of animal vocalizations into distinct behaviorally relevant groups for communication is an essential operation that must be performed by the auditory system. This auditory object recognition is a difficult task that requires selectivity to the group identifying acoustic features and invariance to renditions within each group. We find that small ensembles of auditory neurons in the forebrain of a social songbird can code the bird’s entire vocal repertoire (~10 call types). Ensemble neural discrimination is not, however, correlated with single unit selectivity, but instead with how well the joint single unit tunings to characteristic spectro-temporal modulations span the acoustic subspace optimized for the discrimination of call types. Thus, akin to face recognition in the visual system, call type recognition in the auditory system is based on a sparse code representing a small number of high-level features and not on highly selective grandmother neurons.

## In brief

To translate communication sounds into meaningful messages (call types in animals), the auditory system needs to identify the acoustic features characterizing the messages and ignore individual utterance variations. Robotka et al. show how this sound categorization task is performed in the avian auditory cortex by small neuronal ensembles.

## Graphical abstract

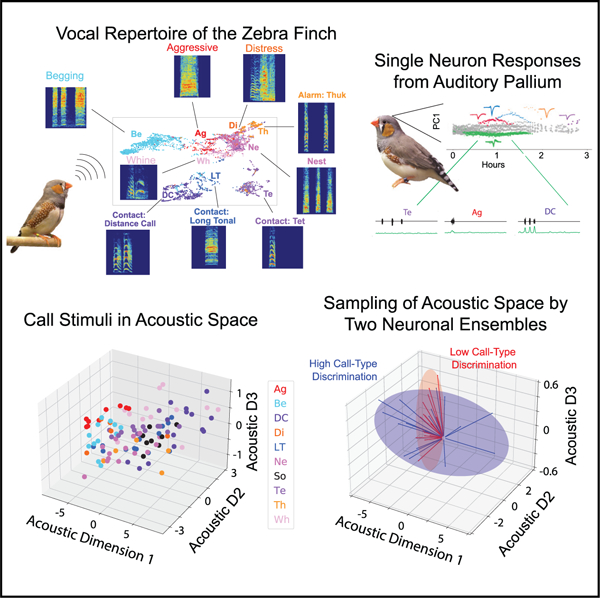

## INTRODUCTION

The categorization of sensory stimuli into meaningful objects, whether it be faces by the visual system^1^ or communication calls by the auditory system,^2,3^ can be thought of as the pinnacle of the perceptual computations performed by the brain. Auditory categorizations are required for the sound to meaning transformations and these are essential for animal vocal communication and human language. Auditory categorization is a difficult task because it requires partitioning of the continuous acoustic space of meaningful vocal calls and utterances, such as vowel sounds in speech.^4^ Even when the categories are acoustically discrete, auditory categorization requires the neural representation to be robust to several sources of variability^5,6^ such as individual signature, renditions from the same vocalizer,^7,8^ and degradations from sound propagation^9^ or contamination by other sound sources.^10–12^ Thus, auditory categorization of communication calls requires neural tuning for the categories, neural mechanisms for parceling out continuous acoustical spaces, and neural invariance to renditions and distortions within the perceptual categories. Although the mammalian neural auditory circuits that are performing various forms of operations needed for auditory categorization are beginning to be well deciphered^13^ and single neuron selectivity for call categories have been reported,^14–16^ the ensemble neural code used by the mammalian auditory cortex or its avian equivalent, the auditory pallium, to represent an animal’s entire vocal repertoire has not yet been described.

The zebra finch is a well-known model system for the study of the neural basis of vocal production plasticity, which we have more recently been developing to study perceptual mechanisms involved in vocal communication.^17^ In previous work, we analyzed the acoustic code used to distinguish call types and the neural representation of these call types at the level of single neurons. The zebra finch repertoire can be sub-divided by human observers into approximately 10 call types that are used in different behavioral conditions and for different purposes including preserving contact within a group, food begging in juveniles, pair bonding, individual recognition, sounding the alarm, aggression, and mating.^18^ Within each call type, acoustic variations are found across renditions from the same vocalizer, according for instance to the level of stress of the emitter,^19^ as well as across individuals where it is used for identity discrimination by bird receivers.^20^ We described the acoustic code of the ‘‘language’’ of this songbird and showed using supervised classifiers that, despite individual signatures and renditions variations, call types can be discriminated with approximately 65% accuracy across all call types and with most errors occurring for call types found along an acoustic continuum.^21^ In previous research, we also began to investigate how call type can be decoded from the activity of single neurons found in the avian primary and secondary auditory cortex-like regions. We showed, using playbacks in anesthetized animals, that auditory neurons could show a significant degree of selectivity for specific call types and invariance for renditions by different vocalizers within that sound category. Single neurons with high values of selectivity and invariance could efficiently decode particular call types (up to 90% correct classification for a single call type) but the average single neuron decoding performance across all call types is around 30% for the subset of auditory units that had significant information about call type categories.^7^

In this study, we build on that work to investigate the ensemble neural code for call type categories in freely moving animals. Although we found in our previous work that single neurons can efficiently be used to decode a single call type category, decoding the complete repertoire would clearly require multiple neurons. How many? Moreover, our previous investigations had not revealed selective single neurons for all call types. If highly selective neurons are not found for all call types, how are those call types represented in the ensemble neural activity? Finally, we investigated the extent with which the neural representation is shaped by the bird’s behaviorally relevant call type categories beyond what could be expected from the acoustical properties of the sounds. To describe the neural tuning of high-level auditory neurons to these short but complex sounds, we developed a methodology for estimating a neuron’s *invariant* auditory receptive field. First, a neuron’s tuning is described with a modulation power receptive field which permits shifts in time and frequency of the specific acoustic pattern that drives a neural response, and thus accommodates phase invariance akin to the spectral power models used to describe complex visual neurons in V1. Second, for each neuron we estimate multiple modulation receptive fields (MRFs) to capture potentially distinct tuning in the onset versus sustained part of neural response or more generally for temporal spike patterns at different timescales. The same analyses are also performed on a dataset obtained in anesthetized birds and the results are compared (see figures in the supplemental information).

## RESULTS

### Neural recordings in auditory forebrain of zebra finches in response to their complete vocal repertoire

**Figure 1.**
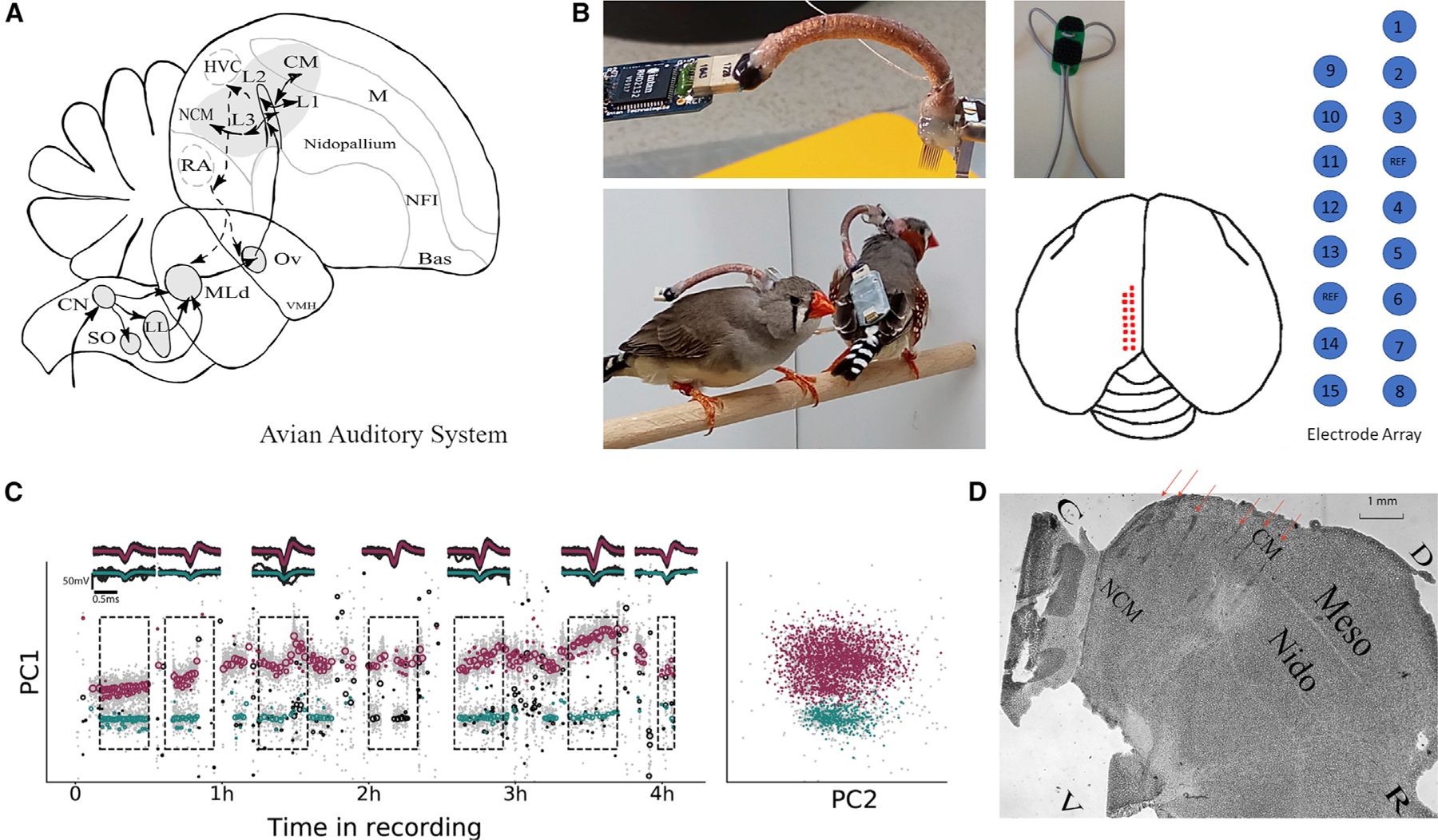
Chronic recording with electrode arrays in freely moving birds (A) Sagittal cartoon of the avian ascending auditory system. (B) Sixteen-channel electrode arrays were inserted in the caudal medial region of the pallium to obtain neural recordings from primary auditory pallial areas, CLM, L1, L2, L3, and secondary auditory areas CMM and NCM. Neural signals were processed using Intan amplifier circuit boards that birds carried on their back. (C) A hierarchical spike sorting algorithm was used to identify spikes from candidate single units across time. Dashed rectangles correspond to the spike clusters above the plot, showing how the spike shapes of two units drifted during the recording but were joined by this procedure. (D) Nissl-stained sagittal slice showing the electrode tracks going through the caudal mesopallium and caudal nidopallium. NCM, nidopallium caudal medial; CM, caudal mesopallium; D, dorsal; V, ventral; R, rostral; C, caudal; Meso or M, mesopallium; Nido, nidopallium. See Elie and Theunissen^17^ for a complete description of the ascending auditory pathways shown in (A).

We obtained neural responses from the auditory forebrain areas of four freely behaving zebra finches and of four urethane anesthetized adult zebra finches (four males and four females) using electrode arrays. The electrode arrays were lowered in the caudal medial pallium to target the primary auditory cortex-like region, field L of the nidopallium and caudal lateral mesopallium (CLM), and the secondary auditory cortex-like regions, the caudal medial mesopallium (CMM) and nidopallium caudal medial (NCM) (see Figure 1, STAR Methods^,7^).

**Figure 2.**
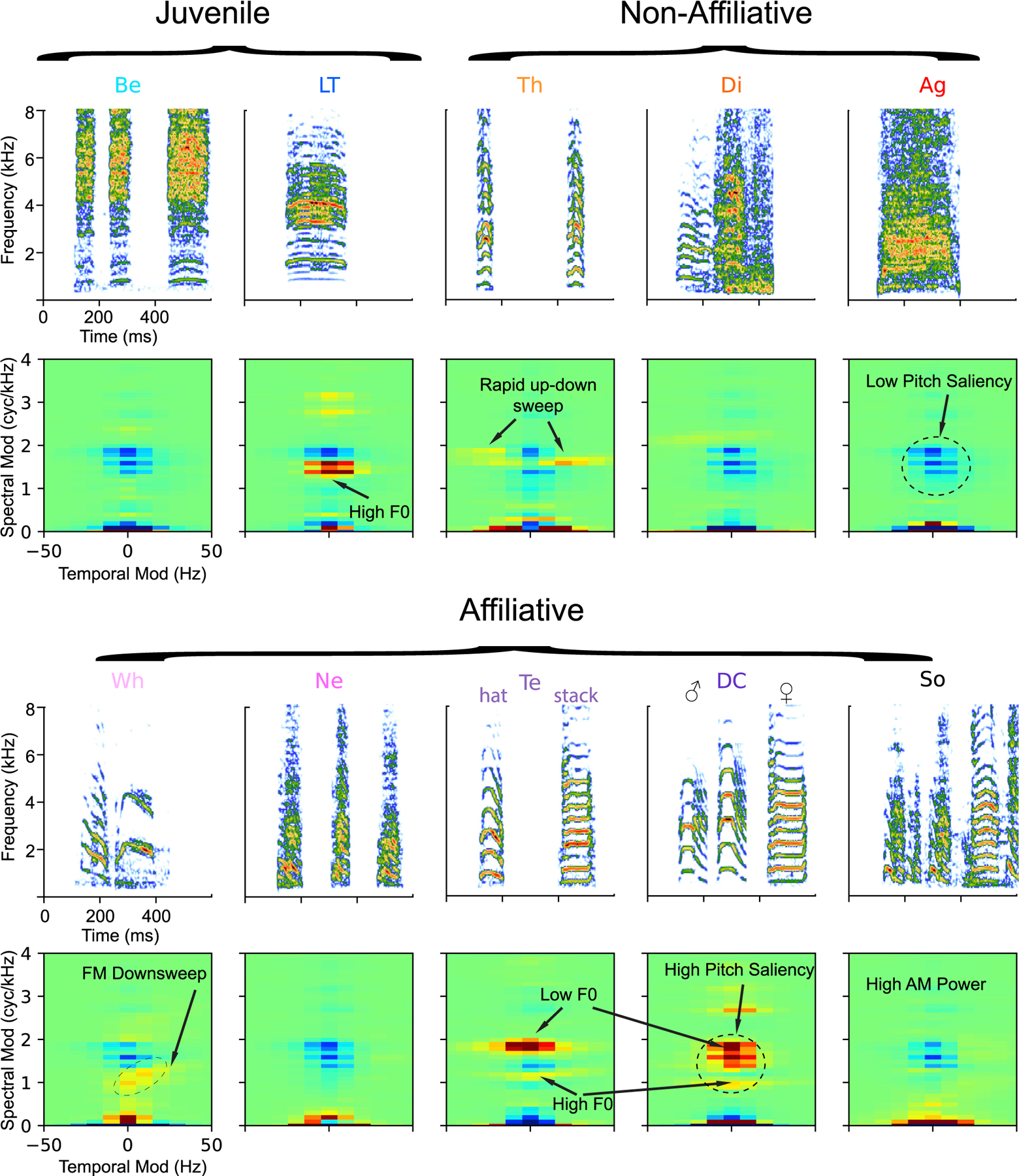
Vocal repertoire in zebra finches Spectrograms (top rows) of example vocalizations and modulation power spectrum (MPS) deviations (bottom rows) for each of the 10 ethogram-based call types of the zebra finch vocal repertoire. Be, begging; LT, long tonal; Th, Thuk; Di, distress; Ag, aggressive; Wh, whine; Ne, nest; Te, Tets; DC, distance call; So, the male song. Song was cut at 600 ms to match the timescale used to display all calls. The Te call category includes hats and stacks and the DC is sexually dimorphic. The MPS deviations are the difference between the average MPS of a given call type used in these experiments (Be: n = 12; LT: n = 13; Th: n = 5; Di: n = 5; Ag: n = 9; Wh: n = 13; Ne: n = 15; Te: n = 15, DC: n = 14, So: n = 9) and the average MPS across all call types (n = 110). This repertoire average MPS and example MPS deviations for single renditions are shown in Figure S1. Red, above average power; blue, below average power.

While recording neural activity, we played back distinct renditions (5–15, mean = 11) of 10 call types from the zebra finch vocal repertoire (110 stimuli and up to 10 trials for each). The 10 call types, as defined by human observers based on behavioral context of call occurrence and perceptual call similarity (ethogram-based call types),^21^ include the two juvenile calls, the begging call (Be) and the juvenile contact call named long tonal (LT) call; three non-affiliative calls, the alarm call named Thuk (Th), the distress call (Di), and the aggressive call (Ag) also called Wsst; and five affiliative calls, the nest (Ne), and whine (Wh) calls made as part of the pair-bonding and nest-building behavior, the soft contact call named the Tet (Te), the sexually dimorphic loud contact call named the distance call (DC) and the song (So) produced only by the male zebra finch as part of courtship behavior (see example spectrograms in Figure 2^21^). Song is considered as one of the categories of vocal communication signals in the zebra finch repertoire and here referred to as one of the ethogram-based call types without any reference to the learn or innate aspects of production. The Te calls could also be further subdivided into so-called stack calls and hat calls^18,22^ but are lumped in the Te category in this study. Acoustical analyses performed in Elie and Theunissen^21^ and as part of this study (see below and Figure 6A) show that these ethogram-based call types are found both in separate discrete regions of the physical acoustical space as well as in a continuum; just as humans, zebra finches use both discrete and graded signals for vocal communication.

### Auditory units have heterogeneous and selective responses

**Figure 3.**
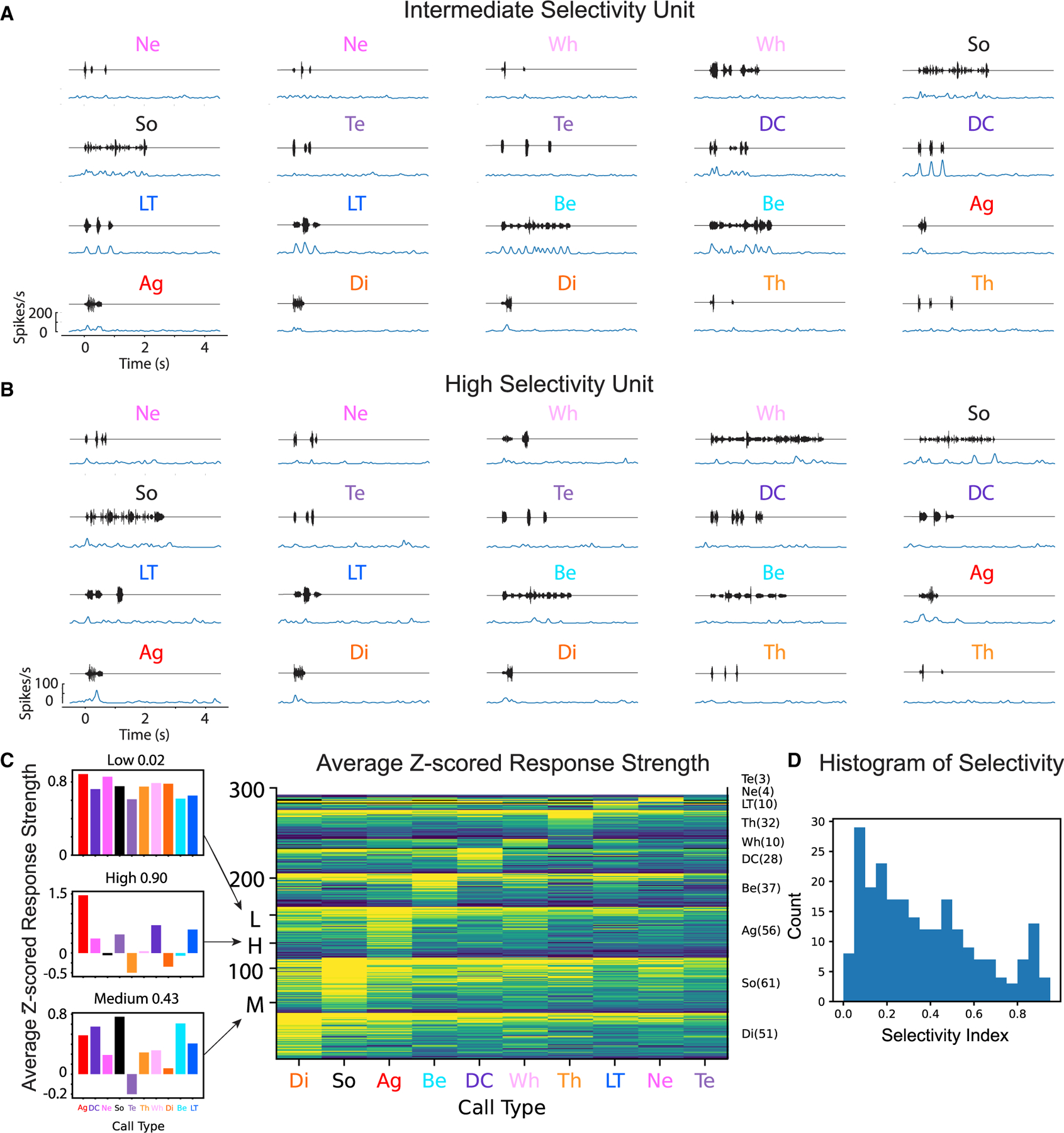
Neural responses to repertoire and selectivity (A and B) Time-varying average neural responses (PSTH) of one intermediate selectivity and one high selectivity auditory unit. For each example call, the top line is the oscillogram of the sound that was played back. The bottom blue line is the smoothed (30 ms window) PSTH. Example spike rasters are shown in Figure S3. Selectivity metrics and decoding estimation were based on responses obtained for all 110 stimuli, but only 20 stimuli are shown here. (C) Average responses strength to each call type for three example units with low, medium, and high selectivity. The title of each bar plot shows the value of the Z-based selectivity index (SI) for the corresponding example unit. The *Z* scored response strength (Z) of all auditory units in the entire dataset (n = 292) is shown on the color matrix. Each row of this matrix shows the average Z obtained in a unit for each call type of the zebra finch repertoire. The call types on the x axis have been sorted according to the average Z for that call type across all units: distress calls (Di) had the highest average response strength followed by song (So), etc. Units on the y axis are first sorted according to the call type that gave the highest Z for that unit following the order of the x axis: 51 units had the peak responses for distress call, 61 for song, etc. Within each of these blocks, the rows were further sorted according to the Z-based SI: the most selective units are on the bottom of the block and the least selective at the top. (D) Histogram of the Z-based SI for the 292 units shown in (C).

Our spike-sorting procedure allowed us to extract putative single units and we chose to select a subset of those units using a signal-to-noise ratio (SNR) threshold of 5 (Table 1). Based on the examination of the refractory period, we believe that the majority of these units are single units, but not all of them (see STAR Methods). We refer to these high SNR clusters as units, with the understanding that some of them might correspond to two (or more) neurons recorded with very similar spike shapes, on the same electrode, either simultaneously or in succession. Auditory units display highly heterogeneous response properties to the various ethogram-based call types with a range of phase locking, response strength, and selectivity for specific vocalizations. Figure 3 shows example responses of two units from the awake dataset, one exhibiting an intermediate level of selectivity (Figures 3A and S3) responding well to DC, LT, and Be calls and one being highly selective, exhibiting a stronger preference for the Ag call (Figure 3B). A range of response strength and selectivity is also observed within primary and secondary areas within the auditory pallium (Figure S5) (Video S1). We described a similar heterogeneous set of responses in the anesthetized preparation, which also exhibited varying degrees of selectivity (see Figure 1 in Elie and Theunissen^7^).

To visualize the selectivity for ethogram-based call types across all neurons, we generated a color matrix of the *Z* scored spike rate response relative to background (*Z* scored response strength *Z*, Figure 3C, see STAR Methods). Each row of this matrix corresponds to an auditory unit. The rows are first arranged in blocks according to the call type eliciting the highest response strength and then further sorted within that block according to the degree of selectivity for that call type as measured by the *Z*-based selectivity index (see STAR Methods). These neural response matrices show that, based on spike rates only, there is a range of tuning in terms of preference (highest response strength) with a bias toward the longer calls (Ag, Be, So, and Di), as well as a range of selectivity both overall and within each block. Nonetheless, it is clear from this pictorial representation that call type could be decoded from the ensemble response: the bands of higher activity seen in the ‘‘diagonal’’ are a clear indication of the tuning for call type. Very similar results are observed in the larger anesthetized dataset (Figure S2).

### Decoding call types from ensemble responses

**Figure 4.**
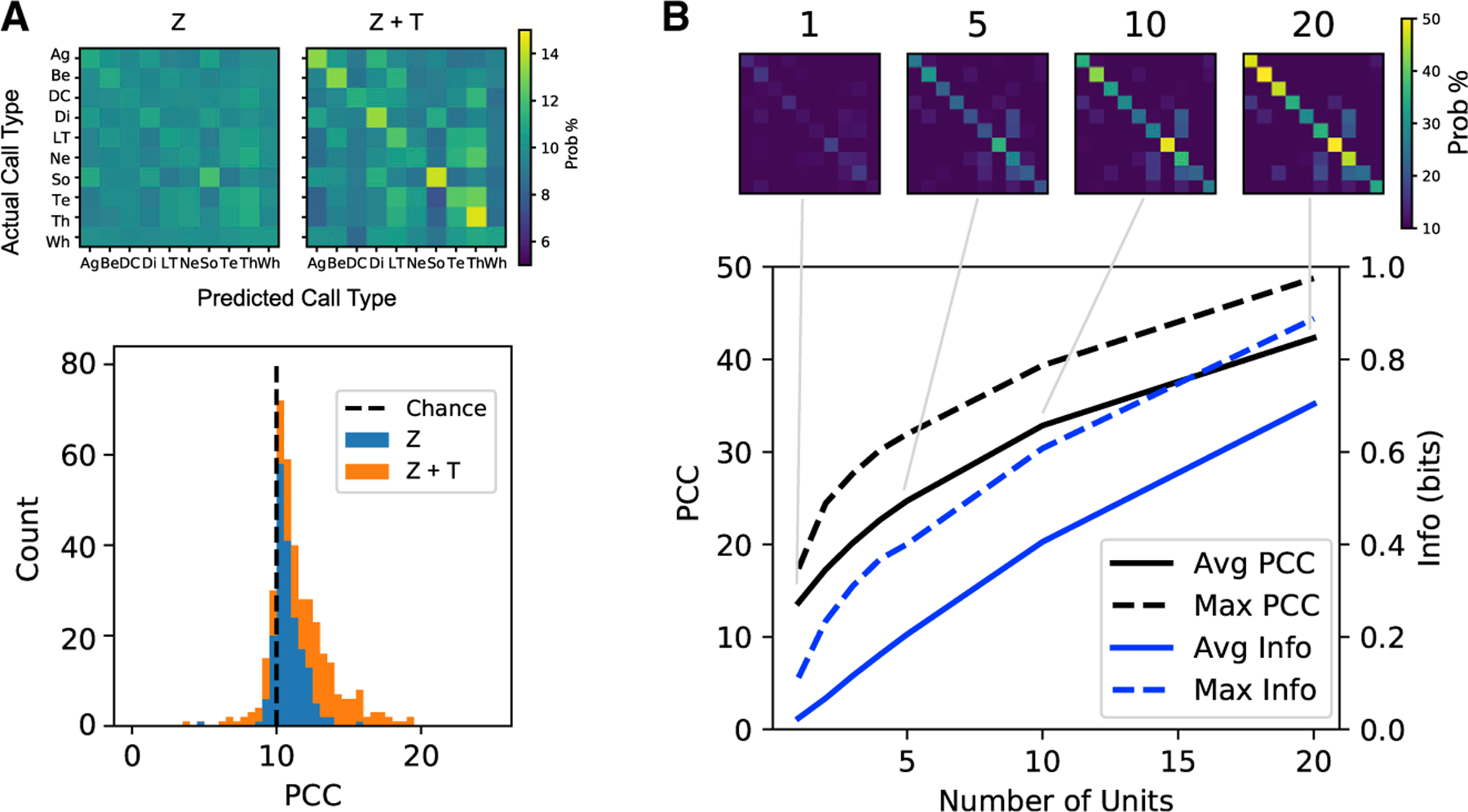
Decoder performance (A) Unitarian decoder: average confusion matrices (top row) and stacked histogram (bottom panel) of the percentage of correct classification (PCC) for a naive Gaussian Bayes decoder trained independently for each unit (n = 191) to classify call type using only the response strength (Z) or the response strength and temporal patterns (Z + T). The PCC is the average of the diagonal in the confusion matrix. Chance level is 10%. (B) Ensemble decoder: average confusion matrices (top row) and decoder performance (bottom panel) as a function of the number of units in the decoder. The order of call types in the confusion matrices is the same as in the confusion matrices in (A). The solid black line in the bottom plot is the average PCC_Z+T_ estimated over 10,000 ensembles obtained by sampling from 100 neurons (for ensembles of 1 and 2, only 100 and 4,950 different ensembles can be sampled). The blue lines quantify the decoder performance by estimating the mutual information from the confusion matrices as in Elie and Theunissen^7^ (right y axis). The dashed lines are the performance of the neuronal ensembles in the top 10%. Note the differences of the probability scale between (A) and (B). Corrected standard errors of the mean PCC ranged between 0.24%, for ensembles of 1 unit, and 1.17% for ensembles of 20 units and are not shown on the graph.

To quantify the decoding of ethogram-based call types, we fitted a naive Bayes decoder algorithm that used the neural response as its input. For the neural response, we tested a code based on the average response strength *Z* (*Z* scored spike rate with reference to background) as well as a code that considers both the strength (Z) and the temporal patterns (T) of the response. To resolve the problem of high dimensionality of the temporal patterns, we reduced it to the first five principal component coefficients (T_1_, … T_5_) of a principal-component analysis applied to the time-varying response of all neurons to all vocalizations. As shown in Figure S7, these principal components (T) will effectively capture relative weight in the onset versus sustained responses as well as potential offset responses. The use of a neural code based on Z + T clearly boosts the decoder performance relative to just the use of Z, as shown in Figure 4A where the decoder was independently trained and tested with each unit responses (mean percentage of correct classification [PCC]: PCC_Z_ = 10.78%; PCC_Z+T_ = 12.3%; 14% increase; t_(190)_ = 10.06; p < 10^−5^, n = 191 units for which we had data from all 110 calls).

**Figure 5.**
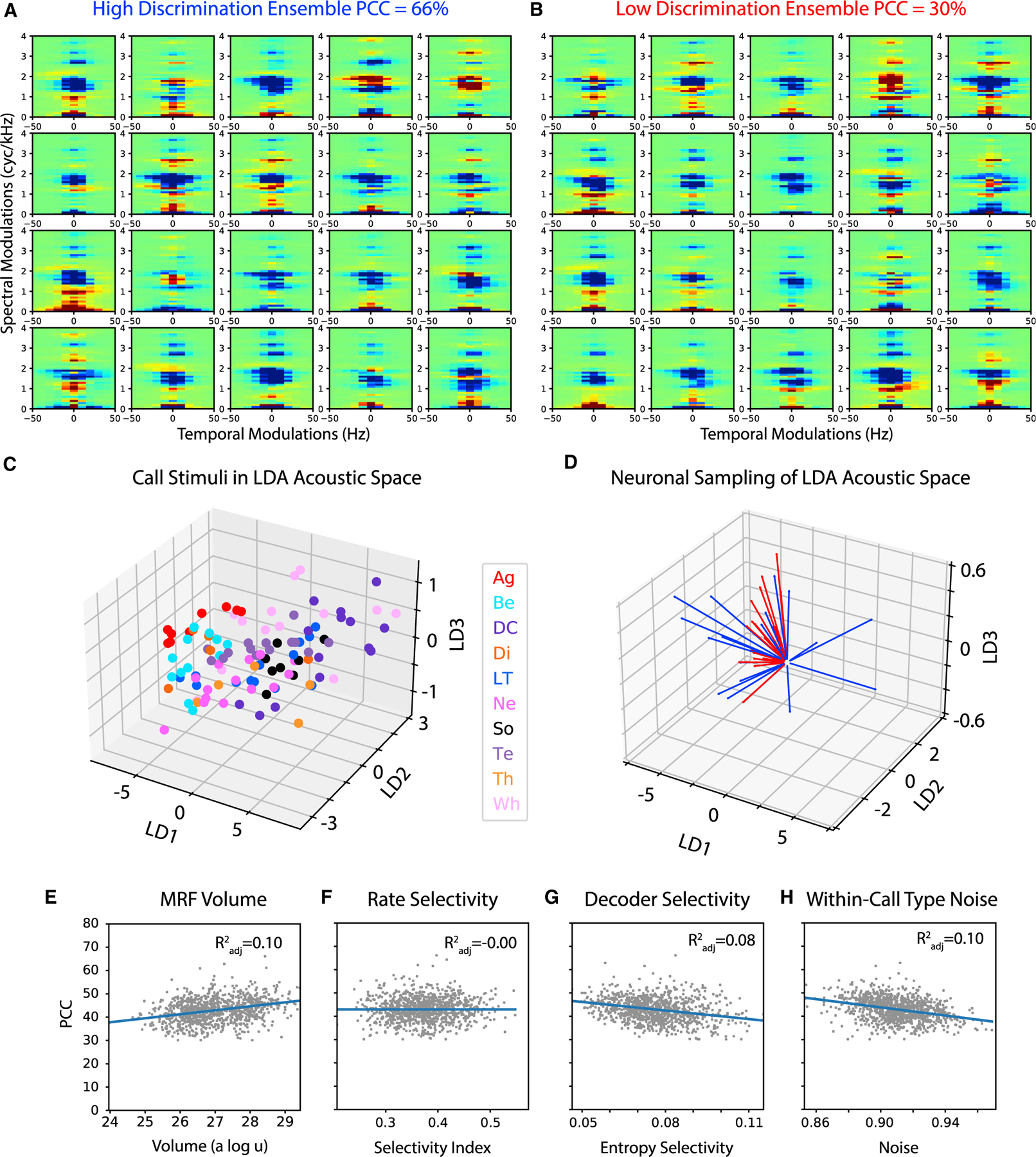
Ensemble neural tuning and decoder performance (A and B) First component of the modulation receptive fields (MRFs) obtained from a highly discriminating ensemble (A) and a low discriminating ensemble (B) each composed of 20 units from the awake dataset. The MRF components shown here are those obtained from the neural response strength Z (MRF_Z_). The other five MRF components of each unit are obtained for each PC of the time-varying response T as shown in Figure S7 for other units. (C) Projection of the MPS of the stimuli used in this experiment in the 3D space spanned by the first three linear discriminant (LD) functions. (D) The MRF_Z_ values shown in (A) and (B) projected as vectors in the same linear discriminant space as in (C): 20 blue vectors correspond to the 20 MRF_Z_ values shown in (A) for the highly discriminating unit and similarly for the 20 red vectors and the MRF_Z_ values in (B). (E–H) Bivariate regression analyses for random ensembles of 20 units (n = 1,746) to analyze the relationship between tuning properties of the ensemble and the discrimination performance measured by the PCC. Each point in these scatterplots corresponds to an ensemble of 20 units. (E) For each ensemble, the volume spanned by all six MRF components (Z + T) of all units in the ensemble. (F) The average SI of the units in each ensemble: this SI, calculated from the response strength Z ranges between 0 and 1. (G) The average entropy selectivity over all units in the ensemble: this index also ranges between 0 and 1 and is based on the PCC of the decoder, which takes into account temporal patterns in the spike trains (see STAR Methods). For (H), the noise is the average of the standard deviation of the *Z* score response strength across all trials and vocalizations belonging to the same call type. Note that if the *Z* score response strength from all vocalizations were used to calculate this standard deviation, this number would be exactly 1. A noise value smaller than 1 indicates higher invariance of neural responses within call types. The coefficient of determination $\left({\text{R}}^{2}{\;}_{\text{adj}}\right)$ is significantly different from zero for (E) (F_(1,79)_ = 8.975, p = 0.0036), (G) (F_(1,79)_ = 6.73, p = 0.011), and (H) (F_(1,79)_ = 8.75, p = 0.004)

To test the decoding performance of synthetic neuronal ensembles of increasing size, we sampled from all auditory units in our dataset (i.e., across all birds and auditory regions) that individually had an average PCC (PCC_Z+T_) above 12% (10% is chance level). The number of units that satisfied that requirement is exactly n = 100. As expected, as the ensemble size grew, the neuronal decoding performance using both the strength and the temporal patterns of the response (Z + T) increases, reaching average levels of 42% for 20 neurons. Data size limitations prevented us from fitting and testing decoders for larger ensembles (see STAR Methods). The top 10% of the neuronal ensembles of size 20 reach an average PCC_Z+T_ of ~50% (Figure 4B) and the best ensembles (e.g., Figure 5A) reach values close to the optimal performance of 65% that we had achieved in previous supervised classification of call types using acoustical features.^21^ The average decoding performance based solely on the response strength (Z) is around 30% for neuronal ensembles of size 20, showing that the temporal patterns in the response carry information not only at the unit level but also for ensemble codes (mean PCC_Z_ = 29.6%; mean PCC_Z+T_ = 42.3%; 43% increase; t_(160)_ = 8.83, p < 10^−5^). The average decoding performance in the anesthetized dataset is slightly better (Figure S8). This increase in performance under anesthesia can be explained by a greater SNR in the neural response. This higher SNR is made evident in the average time-varying rate that shows lower background rates relative to peak rates (compare average rates on the left and right panels in Figure S7A) and lower neural noise during the response (compare Figure 5H with Figure S8H) in the anesthetized dataset.

An examination of the confusion matrices (Figure 4B) shows that most misclassifications originated from the same systematic errors: the soft Ne call is often confused with the soft contact Te call; the juvenile LT call with the Be call; the alarm Tu call with the Te call; and the Di call with the Ag call. Similar systematic errors were found in the acoustic decoding (see Figure 10 in Elie and Theunissen^21^). Some of these systematic misclassifications have a biological explanation. The Te call is a soft contact call that can sometimes be produced in the same context as Ne calls, which are soft vocalizations produced for social bonding when birds are interacting very closely to each other.^18^ In these situations, other physical behaviors would further define the behavioral meaning of the vocalization. The juvenile Be call matures into the LT contact call and then into the adult DC, forming an acoustic continuum^23^; Di and Ag calls make an acoustic continuum characteristic of a graded signaling system.^21^

Given that we are estimating these confusion matrices for ensembles up to 20 neurons but from a limited sample of 100 neurons, the statistical robustness of these results must be assessed. First, note that all of our measures of standard error and the corresponding inferential statistical analyses are based on a sample size of n = 100 and not based on the 10,000 permutations that we tested (see the statistical corrections required for these ensemble measures in STAR Methods: statistical analyses). Second, the validity of these results are supported by multiple ‘‘biological’’ cross-validations: we obtain very similar results both in terms of average decoding performance and systematic error when this ensemble decoding analysis is performed in the anesthetized dataset (Figure S4); separately for each bird (Figure S4); for units sampled solely from primary (field L/CLM complex) versus secondary (NCM and CMM) avian auditory pallial regions (Figure S6); across male and female birds (Figure S4); and a similar increase in decoding performance as a function of the number of neurons is obtained in the primary and secondary auditory pallium (Figure S6). Note, however, that because the firing rate is significantly lower in the secondary auditory pallium compared to the primary auditory pallium in the anesthetized dataset, decoding performance on a ‘‘per spike’’ basis is higher in secondary regions (Figure S6).

Thus, the activity from relatively small ensembles of auditory pallial neurons sampled either from the primary or secondary auditory areas (or both) can be used to identify the ethogram-based call types, or in other words, the meaning of any vocalization.

### Modulation receptive fields of single neurons and ensemble decoding performance

To further understand how these neuronal ensembles achieve their decoding performance, we investigated the tuning of neurons in these ensembles. For this purpose, a representation of the sound that reveals discriminative features between ethogram-based call types was highly desirable. Whether they are found in distinct acoustic groups or along a continuum, call types are primarily distinguishable by spectral features that describe their pitch saliency and the frequency of their formants. For example, as one can see on the example spectrograms of Figure 2, the contact calls (Te and DC) have very high pitch saliency (reflected in their tonal sound quality), whereas the Ag and Be calls have low-pitch saliency (reflected in their noisy sound quality). The formants are regions in the frequency spectrum that are emphasized by the filtering properties of the upper vocal tract. Birds, just like humans, can shape their upper vocal tract to change the frequency of these resonances.^24,25^ The Be calls have a high formant band above 4 kHz, whereas Ag calls have a lower formant frequency band centered at around 2 kHz. The duration, loudness, and to some extent the fundamental frequency (pitch), can also be used to determine the call type. In addition to such predefined bioacoustical measures, we also used as features in our classifiers the spectrogram and the modulation power spectrum (MPS).^21^ They both have the advantage of being assumption-free regarding the coding of acoustic features, and yielded similar classification performances, albeit slightly lower, 60% and 55%, respectively, versus 64% obtained using bioacoustical measures (see Figure 10 in Elie and Theunissen^21^). To analyze the ensemble tuning responses, we chose the MPS representation. The MPS is obtained from the 2D Fourier transform of the spectrogram and is very well suited to characterize the spectral and temporal features that one can observe on a spectrogram, such as pitch saliency, formants, rates of amplitude modulation, and the fundamental frequency (Figures 2 and S1^,26,27^). Moreover, while these acoustical characteristic features can be found at any time point in a spectrographic representation, thus hindering any possible averaging, the same features will always be found at the same spectral-temporal modulation location in the MPS, allowing us to obtain averaged MPS for any group of sounds (e.g., a call type or all vocalizations) as well as neural response-weighted MPS to characterize a neural tuning that might be invariant to small time and frequency shifts in otherwise characteristic sound features. We call the neural response-weighted MPS the MRF. Given its time-invariant property, the average MPS obtained for a call type reflects what is observed across all renditions of that call type, as opposed to the average spectrogram which would blur out the features (Figure S1). For example, in the MPS of zebra finch vocalizations, the presence or lack of energy in the spectral modulations found between 1 and 2 cycles/kHz distinguishes high versus low pitch saliency calls (i.e., tonal versus noisy); the low pitch of the harmonic stack in the Te (around 550–700 Hz) is reflected in the excess power just below 2 cycles/kHz, whereas the higher pitch LT (around 700 Hz) shows excess power around 1.4 cycles/kHz; and the relative slow frequency downsweeps often present in the Wh are reflected as power around 1 cycle/kHz (1,000 Hz pitch) but with positive temporal modulations (Figure 2).

An MRF can be estimated by a spike-triggered average (STA) or, similarly, by a neural response-weighted average (Figure S1). Here, we characterized the tuning of each neuron by estimating six of such weighted averages using the response strength Z and each of the five principal component coefficients describing the time-varying response T. In other words, the tuning of each neuron is characterized by six MRF components, akin to a multi-filter representation that has been used to describe non-linear auditory responses.^28^ Note that the MRFs presented here are not normalized by the stimulus auto-correlations: they are similar to STAs and not to spectro-temporal receptive fields. The MRFs are not-normalized so that they can be compared directly to the discriminant functions obtained in the acoustical analysis as described below. Figure 5 depicts the first MRF component based on the weighted average of Z (MRF_Z_) for neurons belonging to two neuronal ensembles of size 20, one with high decoding performance and one with low decoding performance. As expected, receptive fields of a given ensemble are heterogeneous; a closer examination of Figure 5A shows MRF_Z_ values tuned to low pitch saliency calls (e.g., first row, third column), MRF_Z_ values tuned to high pitch saliency calls (e.g., first row, fifth column), MRF_Z_ values tuned to low pitched tonal calls (e.g., first row, fourth column), etc. Moreover, the tuning obtained with T shows that the temporal patterns can provide both redundant and complementary tuning (see Figure S7); more concretely, the onset versus sustained neural responses can be tuned to different acoustic features, as we have also shown in previous work using an information theoretic approach.^8^

Thinking of the tuning of an ensemble of neurons as spanning an auditory subspace, one is then left with the question of what subspace is being spanned and whether the basis set composed from the responses of individual neurons is indeed a ‘‘good’’ basis set for the task at hand, in this case the representation and thus discrimination of ethogram-based call types. To visualize this basis set in an auditory space that optimizes call type discrimination, we projected the high-dimension MRF components (103 temporal modulation bins × 192 spectral modulation bins = 19,776 dimensions per MRF component) in the reduced space of a Fisher linear discriminant analysis (LDA) trained to best discriminate call types based on the MPS of the vocalizations. The vocalizations in the LDA space are shown in Figure 5C, while the vectors representing each MRF_Z_ from the high and low discriminating ensembles are shown in Figure 5D. Visual inspection of Figure 5C (and Figure S8C) suggests that call types could be acoustically separated in a relatively low-dimensional acoustical space, albeit larger than the three shown here for visualization. Indeed, using LDA on a much larger dataset of vocalizations, we had shown that five dimensions in the MPS space discriminates the 10 ethogram-based call types with an accuracy (PCC) of 55%.^21^ Visual inspection of the MRF_Z_ projected in that same space (Figure 5D) also suggests that the level of call type discrimination that a given ensemble of 20 neurons achieves, scales with the degree to which its ensemble MRFs can span this subspace defined by the LDA. To test this hypothesis, we estimated the volume spanned by each of the ensembles of 20 units in the 3D LDA space and correlated that volume with the PCC obtained from the Bayes naive decoder for that ensemble. Supporting this hypothesis, we find that, as the volume span increases the PCC increases (Figure 5E, $R^{2}{\;}_{\text{adj}}=0.1$, p = 0.0036; Figure S8E for the anesthetized dataset, $R^{2}{\;}_{\text{adj}}=0.48$, p < 10^−5^). The same relationship is found when data are analyzed separately for the primary and secondary auditory pallium (Figure S9). The anesthetized data suggest that the relationship between decoding performance and volume spanned by the MRF tuning might be stronger in the primary auditory pallium ($R^{2}{\;}_{\text{adj}}=0.69$, p < 10^−5^).

Beyond the volume spanned, the actual direction of the MRF vectors could also affect decoding performance. As a proxy for a measure of how close an MRF vector is to a vector perfectly pointing to the centroid of a given call type acoustic feature in the 3D LDA space (as shown in Figure 5C), we used two measures of single neuron selectivity: the selectivity index based on response strength Z (used above and shown in Figures 3C and 3D) and the entropy selectivity. The latter measure is based on the diagonal of the confusion matrix obtained by applying the naive Bayes decoder to both the strength and the time-varying pattern of a unit neural response (Z + T; see STAR Methods). Surprisingly, the average Z-based selectivity index (average across the 20 units in each ensemble) is not related to the ensemble decoder performance (Figure 5F), whereas the unit average entropy selectivity is negatively correlated (Figure 5G). This unexpected negative correlation can be explained by the fact that very selective units are only found for certain call types and therefore that ensembles with higher average selectivity also have higher redundancy in their neural tuning. In other words, MRF vectors for these ensembles point to similar directions in the acoustic space.

Finally, we quantified the effect of the neural variability on the decoding performance. The variability in the neural response is not pictorially represented in Figure 5D but can be imagined as oval clouds of responses along each vector. For this purpose, we calculated the variance of the response strength Z within each call type and averaged values over call types and units belonging to the same ensemble. Note that this measure of neural variability includes the neural noise observed from trial to trial for the exact same sound stimulus as well as the variability in the time-varying mean response to different exemplars of the same call type (i.e., the lack of invariance). As shown in Figure 5H, as neural variability (neural noise + lack of invariance within call type) increases, the decoder performance decreases ($R^{2}{\;}_{\text{adj}}=0.1$, p = 0.004).

In conclusion, small ensembles of auditory pallial neurons can efficiently encode the ethogram-based call type. This efficiency is greater when the tuning responses maximally span the acoustic subspace that is occupied by these natural vocalizations and that maximizes the distance between call types (as given by the LDA). This efficiency is also greater for neural responses with smaller neural noise and greater invariance to acoustic variability within call types. However, decoder performance is not correlated with unit selectivity: the basis set from the tuning responses does not have to ‘‘line-up’’ (and does not) with a basis set where each vector points toward one call type. When all four regressors are taken into account (i.e., volume spanned, neural variability, and the two measures of selectivity) in a linear model to predict PCC, one obtains a coefficient of determination $\left(R^{2}{\;}_{\text{adj}}\right)$ of 0.22 (F_(4,76)_ = 5.832, p = 0.00038). Note that the coefficient of determination quantifies the fraction of the total variance (and not the explainable variance) of the decoder performance that is explained by the regressors (the tuning properties). Because the naive Bayes decoder is trained and tested in cross-validation on single trials data, the non-explainable variance of the decoder performance corresponds to variations in the neural responses between presentations of the same exact sound stimulus. This variability could be large, in particular for the awake dataset where the state variability of the subject yielded noisier responses. Our single measure of neural noise captures some of that variability but not all of it. In the anesthetized dataset, the fraction of explainable variance relative to the total variance is much higher because the variability in the responses is smaller and thus we obtained more reliable estimates of the decoder performance and of the tuning parameters (Figure S8). Consequently, we find similar results as in the awake dataset, but with much higher effect sizes. In the anesthetized dataset, the effect size (i.e., explained variance of PCC of call type) for the volume spanned by the MRF vectors is $R^{2}{\;}_{\text{adj}}=0.48$ (F_(1,343)_ = 314.8, p < 10^−5^), the effect size for the neural variability is $R^{2}{\;}_{\text{adj}}=0.38$ (F_(1,343)_ = 209.5, p < 10^−5^) and combining them and including the two measures of selectivity in a multiple linear regression enable us to explain the performance of the ensemble decoder (PCC) with an $R^{2}{\;}_{\text{adj}}=0.55$ (F_(2,341)_ = 215.0, p < 10^−5^). When data are analyzed separately for the primary and secondary auditory pallium, the multiple regression that combines volume spanned, neural variability, and the two selectivity measures can explain the performance of the ensemble decoder with an $R^{2}{\;}_{\text{adj}}=0.752$ (F_(4, 125)_ = 98.694, p < 10^−5^) in the primary auditory pallium and $R^{2}{\;}_{\text{adj}}=0.633$ (F_(4,170)_ = 98.696, p < 10^−5^) in the secondary auditory pallium (see Figure S9).

### Neural categories for ethogram-based call types versus acoustical clusters of vocalizations

**Figure 6.**
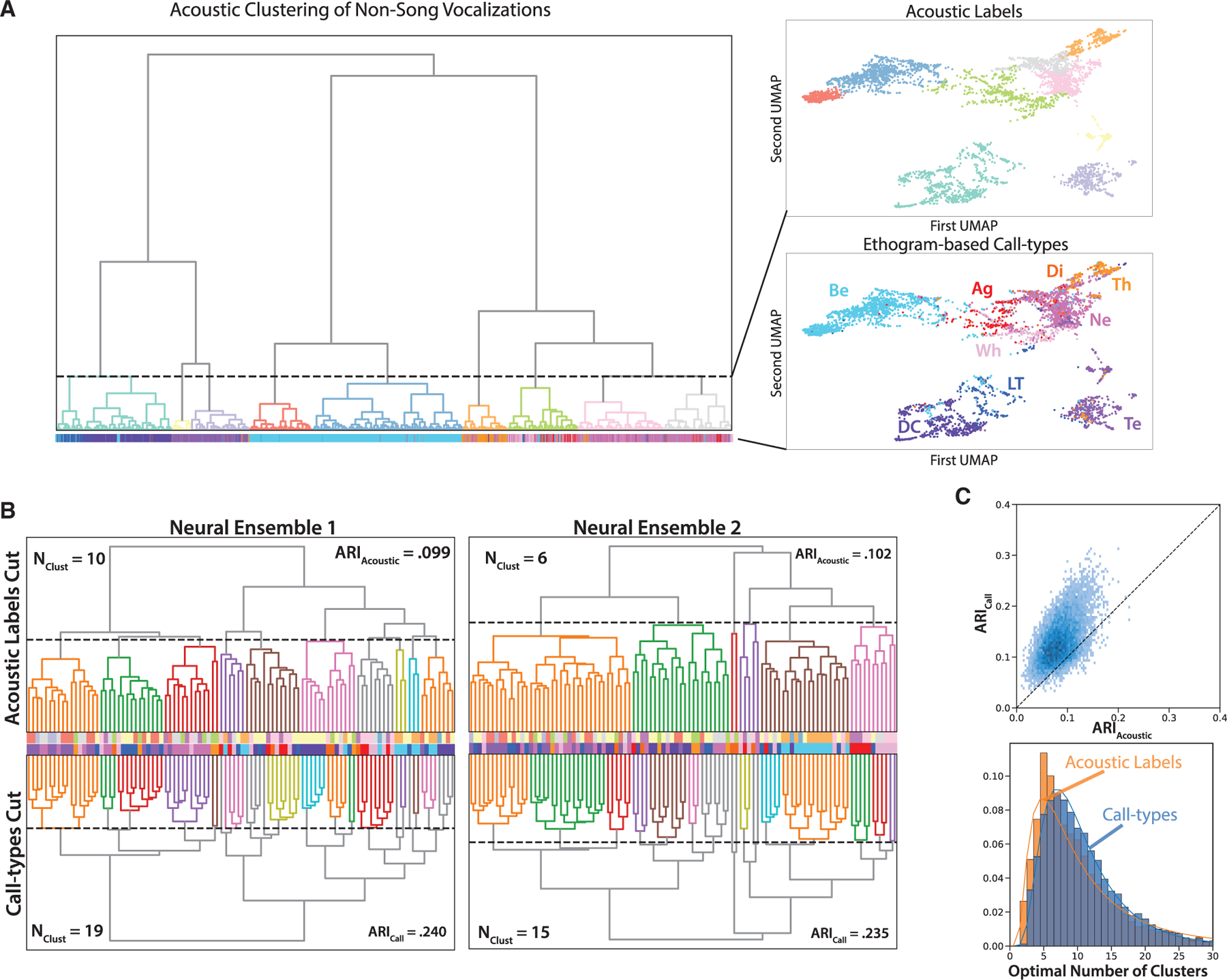
Comparison of acoustic and neural clustering (A) Dendrogram (left) and 2D Uniform Manifold Approximation and Projection (UMAP) projections of vocalizations (right) displaying the results of a hierarchical clustering analysis performed on acoustic features of 4,987 zebra finch calls from the 9 call types excluding song. The colors of the segments in the dendrogram correspond to the acoustic labels also shown on the 2D UMAP projections on the top right panel. The tick marks below the dendrogram are colored according to the ethogram-based call types. This classification is also shown on the same UMAP projection in the bottom right panel. (B) Hierarchical clustering (HAC) for two ensembles (left and right) of 20 neurons. For each neuronal ensemble, the dendrogram from the HAC is cut for maximal adjusted Rand index (ARI) with acoustic labels (upper dendrogram) and ethogram-based call types (lower dendrogram). The tick marks between the dendrograms show the stimuli class label, colored according to the acoustic clusters on the top row and the ethogram-based call types on the bottom row. The colored segments in the dendrogram display the neural clusters. (C) Top: scatterplot of the optimal ARI comparing how well the neural HAC predicts the ethogram-based call types versus the acoustic labels for 10,000 random ensembles of 20 units. Bottom: histogram of the number of clusters of vocalizations given by neural ensembles when their HAC tree is optimally cut to give the best ARI with acoustic labels and ethogram-based call types.

The characteristics of the ensemble neural code described above are fully relaying on a classification of vocalizations based on human observations of the birds’ behaviors. One might wonder if behavioral annotations help uncover the perceptual boundaries that birds apply on their species calls and, in that respect, help achieve a classification that better matches what the brain might be doing, or if, on the other hand, these annotations are generating noise that hinders a categorization that would be purely acoustic based and better rendered by unsupervised clustering based solely on the acoustic properties of the calls. To explore these questions, we performed unsupervised hierarchical clusterings on the entire vocalization database (n = 8,136 calls from 45 birds) and measured the performance of neuron ensembles at classifying vocalizations along these acoustic labels. The sounds were characterized by 20 bioacoustical predefined parameters quantifying their temporal, spectral, and pitch properties as described in Elie and Theunissen^21^ where we also showed the efficacy of this low-dimensional representation for coding ethogram-based call types. The analysis was performed both with and without song because song syllables are quite heterogeneous in their acoustical properties and acoustic information that is present in their sequence is not captured in our acoustical parameters. We generated acoustic labels by cutting the tree obtained in hierarchical clustering at different levels to generate either 9 (without song; Figure 6A) or 10 (with song) acoustic categories to match the number of ethogram-based call types. The tree generated with all vocalizations but song syllables (Figure 6A) demonstrates that there is a correspondence between the ethogram-based call type and the acoustic clusters, but with a fair amount of mixing across the soft-affiliative (Ne, Wh, and Te) and non-affiliative calls (Ag, Di, and Th). The best adjusted Rand index (ARI), that quantifies the correlation between the labels given by the cut of the hierarchical tree and the ethogram-based call types is 0.7, with the optimal cut generating six clusters, and 0.49 with the tree cut generating nine clusters. The fact that a higher ARI is obtained with fewer groups reflects both the hierarchical acoustical structure of call types and their graded quality found in certain subgroups. As one can see on a 2D Uniform Manifold Approximation and Projection in Figure 6A, the acoustic clustering lumps the clouds of the DC and LT calls together and splits the Be and Te call clouds each into two subgroups. These differences between acoustic clustering and the ethogram-based call types support the possibility that the perceptual boundaries used by the birds might not always line up with those corresponding to discrete acoustical clusters.

We then compared the performance of ensembles of 20 units (n = 1,746 ensembles sampled from the same 100 units) at classifying the vocalizations according to the acoustic clusters (9 groups + a 10th label assigned to all songs), to the performance achieved with the ethogram-based call types. The neuronal decoding performance (PCC_Z+T_) reaches only 38.0% for acoustic clusters, below the 42.3% performance previously found for call types (t_(160)_ = 2.71, p = 0.0074). Thus, the classification of vocalizations as obtained from the neuron ensemble response is better matched to human classification based on behavior than to purely acoustic classification of calls.

One could also ask what grouping of vocalizations is optimally performed by neuronal ensembles? Indeed, one could postulate that vocalizations are clustered in the neural response at a different level of granularity than the one offered by ethogram-based call types or acoustic labels, for example by combining some of the affiliative calls (e.g., Ne and Wh) in the same group or, similarly, by combining two or more acoustic labels also in the same group. To address this possibility, we performed unsupervised hierarchical clusterings, this time of the ensemble neural responses. As in our decoding analyses, the neural responses were represented by the response strength Z and the first five principal component coefficients describing the time-varying response [T_1_ … T_5_]. The optimal clustering of the neural responses was measured as the one that gave the best match with the clustering of the sounds given by call types or acoustical labels. Optimality of the match was found by cutting the tree of neural responses at different levels, effectively testing different levels of granularity, and calculating the ARI with ethogram-based call types or acoustic labels. Figure 6A illustrates this process for two neuronal ensembles of 20 neurons. Neuronal ensemble 1 exhibits an optimal clustering of 10 groups to obtain the best match with acoustic labels and 19 groups for the best match with the call type labels. Neuronal ensemble 2 exhibits an optimal clustering of 6 groups for acoustic clustering and 15 groups for call types. While the level of granularity (optimal number of clusters of the neural responses, Figure 6C, bottom) can vary widely from one neuron ensemble to the other (range 1–30 clusters), both the median and the average number of clusters are very close to the number of ethogram-based call types irrespective if the acoustic labels or the call types are used as labels (call types, mean = 10.83, median = 9; acoustic labels, mean = 10.03, median = 9; paired t test on log transformed data t_(80)_ = −0.808, p = 0.21). These results indicate that, while some ensembles are lumping or splitting some call categories, the large majority group vocalizations along the same number of categories as obtained by human annotations. Furthermore, in the large majority of all sampled ensembles, there is a better match between the neural clustering with the call type labels than with the acoustic labels (Figures 6B and 6C, top, paired t test t_(80)_ = 5.144, p < 10^−5^). The effect is also shown to become stronger as the size of the neural ensemble increases (Figure S10). Thus, the neural responses alone reveal a hierarchical organization of vocalizations by the auditory brain that is better matched to ethogram-based classification of calls than acoustically driven classification. In summary, the efficient neural coding found at the ensemble level for the ethogram-based call types reflects in part a specialization (potentially learned) of avian auditory pallial areas for grouping the birds’ vocalizations in behavioral meaningful categories.

## DISCUSSION

We find that neurons in the avian auditory pallium of the zebra finch have sufficient selectivity in the acoustic space spanned by call types and sufficient invariance to renditions within a call type to represent most of the calls in the bird’s repertoire. More precisely, the neural activity of small ensembles of neurons (~20) can be used to determine the call type of the animal’s repertoire with a performance (~50% correct classification for 10 call categories for the top 10% ensembles) that is very similar to the one achievable with machine decoders using the acoustic features of the sound.^21^ While one could argue that the performance is far from 100%, it should also be noted that most of the errors are systematic misclassifications among semantically related call types, such as those used in aggression and distress, or among soft call types produced in closed visual contact. Just as humans use linguistic context to interpret homonyms, birds are clearly able to use other sources of sensory information and behavioral context to further interpret meaning of the conspecific vocalizations they hear.

### A sparse ensemble code where the unit selectivity is not aligned with call type

We next examined the nature of this ensemble neural code. We first confirm the finding that single auditory units in the avian pallium have heterogeneous properties^29^ whether those are assessed by their selectivity for particular call types (Figures 3, S2, and S5) or by receptive fields (Figure 5). We also find small differences between the different regions of the auditory pallium. While the selectivity would be slightly higher in secondary areas according to the Z-based selectivity index, when the temporal pattern in the response is taken into account, the trend is reversed: the entropy selectivity calculated from the decoder classification performance is higher in more primary auditory areas (Figure S5) (Video S1). Differences in tuning properties across sub-regions of the auditory pallium have been extensively described by us and others previously.^6–8,12,30–34^ It should be noted, however, that both here and in most of that previous work, the effect sizes are relatively small due in part to the heterogeneity of response properties within sub-regions. This heterogeneity in responses, which is ignored by examining area-averaged tuning responses, could be key for generating a sparse ensemble code; and, in fact, we show that this heterogeneity allows small ensembles of neurons (only ~20) to fully represent the 10 call types in the zebra finch repertoire. Moreover, and maybe more surprisingly, we find no correlation between the average selectivity of the auditory units in an ensemble and the decoding performance of call types by that ensemble (Figure 5): the selectivity of units measured in the stimulus space of call types is not particularly relevant! In the ancient yet on-going argument as to whether the sensory neural code is distributed^35^ or selective and sparse,^36^ our results would argue that, in these high-level auditory areas, the code is more on the sparse/selective side of the spectrum since the activity of a very small number of neurons is sufficient to represent call type identity. However, the tuning selectivity at the level of single neurons does not need to be exactly aligned along the acoustic dimension that would correspond to a canonical call type. In other words, the auditory version of the Jennifer Anniston neuron (also known as the iconic ‘‘grand-mother’’ cell)^37^ or the ‘‘concept cell’’^38^ does not need to exist in these high-level auditory areas; neuronal ensembles are made of cells that are selective for abstract features that make out the auditory objects but their tuning direction does not line up perfectly with uniquely defining features of each call type. As long as the relevant acoustic subspace is well spanned by neurons selective for particular high-level features, call types can be decoded (Figure 5). For these reasons, we propose that a measure of volume spanned of the behaviorally relevant stimulus subspace should replace measures of single-neuron selectivity for assessing tuning selectivity in neuronal ensembles: neuronal ensembles performing categorization are selective for a subset of the stimulus space that is the most appropriate for discriminating the categories but individual neurons (i.e., the ‘‘vectors’’ that span that subset) are not necessarily selective for individual categories. This central conclusion of our study is reminiscent of the recent findings in high-level visual areas sensitive for faces^39^ where neurons are sensitive to high-level visual features called face-patches that can be used efficiently (i.e., using a small number of neurons) for individual face recognition. We postulate that there is no need for ‘‘going all the way’’ to the level of selectivity for single call types (or a single face in the visual domain) and that memory, decision making or motor areas can selectively interpret the output of a particular small ensemble of neurons from high-level sensory areas to generate the appropriate neuronal or behavioral response.

### Advantages of the observed ensemble code

A coding scheme that is based on ensembles of neurons that do not individually encode a single object, but rather abstract features, may be particularly suited to producing invariant representations for the object’s variable perceptual signal.^40^ This encoding scheme may also facilitate other sensory tasks; in the auditory domain, this could include sound source separation^2^ or the memory formation as required for individual recognition.^20,41^ Computational modeling could reveal whether this coding scheme is indeed efficient or optimal for categorical representations of auditory objects composed of a high number of variants. Note that our results do not rule out single neuron selectivity that might be present for learned sound categories in the auditory pallium,^42^ involved, for example, in the recognition of familiar vocalizers such as the bird’s mate. Also, we cannot of course rule out that ‘‘grand-mother’’ cells exist for call categories outside of the auditory pallium and perhaps in the downstream areas but we postulate that they do not, and that such an additional hierarchical processing step is not necessary.

### Readout of the observed ensemble code

The downstream output regions have not been extensively studied in the avian system but could include the song control regions^43,44^; neighboring output regions of the avian forebrain in the arcopallium^17^; the ventromedial hypothalamic nucleus (VMHm), which is part of the social behavior network^45^; or the lateral nidopallium, which has been implicated in higher level cognitive tasks and mate choice.^46,47^

In our ensemble analyses, we combined auditory units recorded from the entire auditory pallium. The auditory pallium of songbirds is a large brain region that has been divided into a primary auditory pallium and a secondary auditory pallium, each of which is further subdivided into subregions^48,49^ that have been compared to different layers of the auditory cortex.^50^ On the one hand, it is improbable, but not impossible, that downstream regions would combine information from both primary and secondary auditory region. On the other hand, either primary or secondary regions could be the source of the downstream information flow. For example, the primary auditory pallium projects to the arcopallium, which could be implicated in generating the appropriate vocal responses or providing feedback to the peripheral auditory system to, for instance, enhance auditory attention to some important sound cues.^17,51,52^ The downstream regions of the secondary auditory pallium have not been examined extensively but it is known, for example, that the ventral NCM projects to the VMHm, which is involved in controlling reproductive behavior.^45^ In our analyses, we also examined the ensemble representation of call categories for units sampled solely from primary or solely from secondary auditory pallial regions (see Figures S6 and S9). We found that in both regions call categories could be represented in the activity of a similarly small number of units: the heterogeneity of the responses is large in both regions and neuron ensembles span the acoustic space occupied by call type categories with the same degree of efficiency. However, there are certainly differences in the representation in these two areas. For example, we found in the anesthetized dataset that the average spiking rate was lower in the secondary auditory areas, yielding an ensemble code that was more efficient on a per-spike basis. Thus, while we observed a similar degree of population sparseness in primary and secondary auditory areas, there is potentially a higher degree of lifetime sparseness in secondary auditory areas. The validation of this result in awake behaving birds and its relevance to downstream information processing will require additional research.

### Multi-component MRFs

Our assessment of a single neuron’s tuning using MRFs enable us to allow for invariance to shifts in time of the spectro-temporal patterns of the sound akin to the use of the Fourier power receptive fields used for modeling high-level visual neurons, such as the complex cells of V1.^53^ We combined this approach with the use of multiple receptive fields to capture a distinct tuning that could be observed for different temporal components of the neural response. As suggested by our previous information theoretic analyses,^8^ the onset and sustained response to these short calls could carry distinct information about call categories. The use of multi-component receptive fields has been shown to be increasingly useful to model auditory neurons in the ascending auditory processing stream,^28,54^ but in prior work these time-invariant multi-component filters were used to model non-linear interactions.^55^ Here, instead, we used multi-component receptive fields to model multiple linear interactions related to distinct temporal aspects of a neural response. This time-varying approach allowed us not only to take into account the temporal patterns observed in the average neural response but also to model time-varying filters relative to stimulus onset.

### Ethogram-based call types versus acoustically defined categories

Finally, we asked whether the efficient code for the ethogram-defined call types could be solely explained by their acoustics. We find that this is not the case because the same number of synthetic groups based solely on acoustical properties are less correlated with the parcellation of the stimuli that can be obtained from ensemble neural responses (Figures 6B and 6C); a result that we also find at the level of single neurons.^7^ These findings suggest that the neural representation is in fact tuned to the categories revealed by the observation of birds’ behaviors around the vocal production, even if these categories sometimes form an acoustic continuum (Figure 6A). It is possible that birds perceive a distinct set of acoustical features than those used in this study and the parcellation of all vocalizations by the birds themselves remains to be determined in controlled behavioral experiments. Nonetheless, the auditory system of the zebra finch is able to form some categorization of call types even when these are found mixed along an acoustic continuum, albeit with some degree of error. This neural coding could implement a form of categorical perceptions akin to what is required for vowel perception in human speech. It remains to be seen whether this neural representation is then innate and/or the result of experience with the vocal repertoire.^56–58^

### Limitations of the study

We found that neural responses were more variable in the awake behaving data than in the anesthetized dataset. It is certain that some of the currently observed un-explainable variance could be explained by analyzing and considering the behavioral state of the birds, for example whether they are attending and responding to the auditory stimuli. We might therefore be underestimating the performance of our neural ensembles and it is possible that an even smaller number of neurons could be used for this call type categorization task.

Note also that we studied the ensemble responses by grouping units that were not recorded simultaneously but could instead be recorded at different sites from the same bird or even from different birds. The use of synthetic ensembles allowed us to explore a much larger number of potential ensembles for a given number of recordings (and thus of birds used). We were, however, unable to consider potential information present in the correlation of simultaneously recorded single trial spike patterns across neurons beyond those expected by the stimulus-driven response. These noise correlations have been shown to exist in the avian auditory system and to be involved in the representation of invariant features of learned acoustical categories.^59,60^ They might also play a role in the representation of call types and a decoder (machine or biological) could exploit them to increase performance. We might therefore again be underestimating the decoder performance.

Furthermore, we have only assessed the ensemble performance using a single type of decoder: a Gaussian naive Bayesian classifier. This classifier yields the solution with maximum a posteriori probability, given that the variability in ensemble neural responses for a given call type follows a multivariate normal distribution. Other machine-based decoders could yield even better results than those reported here. As such, our study most likely calculated conservative lower estimates of the decoder performance.

Finally, we have not investigated the distinct roles of the different sub-areas (akin to cortical layers) or different types of neurons found in the auditory pallium for generating the observed responses. The auditory pallium is a highly interconnected area and the analysis of this microcircuitry for generating the complex responses observed here will be another challenging research project. Given the heterogeneity and overlap in response properties, it is clear, however, that a simple hierarchical feedforward model would be an inadequate model and that, instead, the observed response properties will be understood by examining recurrent network dynamics.

## STAR★METHODS

### RESOURCE AVAILABILITY

#### Lead contact

Further information and requests for data and analysis code should be directed to and will be fulfilled by the lead contact, Frédéric Theunissen (theunissen@berkeley.edu).

#### Materials availability

This study did not generate new unique reagents.

#### Data and code availability

- *Data.* The neural data have been deposited at the NSF sponsored open repository https://crcns.org and are publicly available as of the date of publication. Accession numbers are listed in the key resources table. The neural data in forms of spike arrival times, spike snippets and stimulus files can be found at https://crcns.org/data-sets/aa. The freely behaving dataset is *Avian Auditory 5* and the anesthetized dataset is *Avian Auditory 4.* The repository includes a document that explains the data format. The Avian Auditory 5 dataset comes with a Jupyter Notebook that one can use to browse the entire dataset and generate spike rasters and PSTHs for any unit and stimulus. The raw data files (in the original Intan rhd format) are stored on a sharable google drive and are also available upon request.
- *Code.* All original code has been deposited on the Theunissen Lab github (http://github.com/theunissenlab) and is publicly available as of the date of publication. DOIs are listed in the key resources table.
- Any additional information required to reanalyze the data reported in this work paper is available from the lead contact upon request.

### EXPERIMENTAL MODEL AND SUBJECT DETAILS

#### Ethics *statement*

Animal experiments performed in Seewiesen, Germany, were conducted according to the regulations of the government of Upper Bavaria (Germany protocol number: Az. 55.2-2532.Vet_02-19-131). Animal husbandry or handling was conducted according to the directives 2010/63/EU of the European parliament and of the council of 22 September 2010 on the protection of animals used for scientific purposes. Animal experiments in Berkeley, USA, were approved by the institutional Animal Care and Use Committee (IACUC) of the University of California, Berkeley (US; UCB protocol number: AUP-9157) following the guidelines of NIH and AAALAC.

#### Animals

Experimental zebra finches (*Taeniopygia guttata*) were obtained from our breeding facilities both in Germany (for the awake experiments) and the US (for the anesthetized experiments).

For the awake experiments, two pair-bonded couples (2 male and 2 females total) adult zebra finches were used for the electrophysiological experiments. All birds were 1 year old (between 365 and 730 days) and weighted between 19 and 21g. Prior to the experiment, the birds were raised in a large room size aviary under a 14/10 h light/dark cycle, 24°C, and 30–70% humidity. The approximate number of animals in this aviary is around 100 birds. The birds could physically interact with their colony mates. This allowed for natural vocal, visual and physical interactions with all birds in the colony. During the experiments, the bonded birds were kept in a custom-made, sound-attenuated chamber (sound box). In these experiments, the birds in each pair were implanted in sequence (within a 4 week interval) but always housed together. The birds were separated by a mesh divider inserted in the middle of the sound box when one or both of them were tethered. The mesh partition was transparent and the birds were always able to see each other and interact vocally. When they were not recorded, the birds were freed from their tether and housed together in the same soundbox but without the mesh divider (the detailed timeline is described below). In the soundbox, Zebra finches were kept in a 14/10 h light/dark cycle (fluorescent lamps), 24°C, and 60–70% humidity. This special soundproofed box consists of a plastic-metal standard cage (120 cm × 50 cm × 50 cm), which is placed into a custom-made Faraday cage. The equipment of the box comprised a USB microphone (AT2020USB+, audio-technica), a speaker (FRS 8, 30 w, 8 Ω, VISATON), and a slip-ring miniature commutator (Model: SL-88-10, Dragonfly Inc.) together with RHD2000 ultrathin SPI Interface cables and adaptors (RHD2000 SPI Cable Adapter Board, Intan Technologies) for tethered recordings. As shown in Figure 1., the Intan amplifier circuit board used for filtering, amplifying and digitizing the neural signals was carried on the back of the birds and not on the headstage as it is traditionally done. This setup not only improved the bird’s comfort by decreasing the weight on their head but also allowed them to move more freely by generating torque on the commutators using their entire body weight.

For the anesthetized experiments, we recorded from 4 male and 2 female adult zebra finches but used only data from 2 males and 2 females for the ensemble decoding and encoding analyses performed here. The choice of 4 birds was done by requiring a minimum number of 20 units for which we had responses to 100 common stimuli. Prior to the experimental surgeries and neural recordings, the birds were raised in family cages (112 cm × 56 cm × 36 cm) placed in a ‘‘colony’’ room in the animal facility on a 12.5/11.5 light cycle, 29°C, and 30–70% humidity. Although the birds could only physically interact with their cage mates, the cages were placed to allow for vocal interactions with all birds in the colony and visual and vocal interactions with birds in neighboring cages. For the neurophysiological experiments, the birds were anesthetized with Urethane and head fixed recordings were obtained in a large double walled soundproof box (107 cm × 76 cm × 76 cm) that provides approximately 80 dB of acoustic insulation (Acoustic Systems, Texas). During the recordings, the bird was sleeping inside a bird jacket on a custom heated bird slink with its head facing a speaker (PCxt352; BLaupunkt, Il, USA) placed 40 cm away.

### METHOD DETAILS

#### Implantation of 16 channel multielectrode array and chronic recording of neuronal activity

##### Surgery

For the surgical procedure needed for fixing the head stage onto the skull for the chronic awake recordings, the birds were first injected with Metamizol (100–150 mg/kg, duration ~ 2h) into the chest muscle 30 min before the induction of anesthesia. The birds were then anesthetized using isoflurane inhalation (0.8–1.8% at 0.5L O_2_/min). During anesthesia, the animals were kept warm on a constant temperature heating pad (160 × 160 mm, 12 V/6W, Thermo GmbH) and wrapped in a thin gauze blanket. The subjects were immobilized in a stereotaxic system to maintain their head with an angle of 45° with the vertical. Some feathers of the head were removed, the skin of the head was disinfected. After a subcutaneous injection of 150 μL Procain (Minocain 2%), their scalps were removed. One rectangular opening of 3 mm long and 0.8 mm in width, centered at 0.5 mm lateral in the left hemisphere and 1.25 mm rostral to the Y sinus was created in both layers of the skull and the dura to enable electrode penetration.

A custom design form multielectrode array (MEA) of two rows of 8 platinum-iridium electrodes with parylene C insulation (Microprobe, Custom MEA-PI/Ir with Flexible Cable 16 ch + Ref.; shank diameter 125 μm, tip diameter 1–3 μm, length 5 mm, electrode spacing 250 μm, row spacing 500 μm, Impedance: 4 MΩ) was implanted into the left hemisphere to target the avian auditory cortex. Before penetration, electrodes were coated with DiI powder (D282, Invitrogen, Thermo Fisher Scientific) to enable tracking in histological slices.

One day prior to the implantation we carried out custom-made modifications on the flexible cable of the MEA. The cable was wrapped around tightly with desoldering copper wire (Stannol No-Clean Desoldering Wick, 0.8 mm × 1.5 m – Stannol product Nr.: 870,051) and both ends of the wire were fixed with dental cement (Tetric evoflow, Ivoclar Vivadent GmbH). The silver ground wire was soldered onto the outer surface of the desoldering wire close to the MEA’s side and finally the whole cable was covered with a medical adhesive silicone (Silastic Medical Adhesive Silicone Type A, DOW Corning Corp., USA). The desoldering wire provided an electric shield for the whole 3800 mm length cable and decreased the baseline noise and movement artifacts. The following day we attached the modified MEA onto the custom-made aluminum Microdrive. The aluminum Microdrive has a shuttle with two arms and the epoxy part of the MEA is fixed between these arms with dental cement. The shuttle of the Microdrive slides easily on the aluminum frame driven by a thin screw (1 turn = 250 μm). Before implantation the impedances of the electrodes were measured in physiological saline.

The Microdrive holding the 16 channel MEA was lowered onto the skull surface close to the rostral side of the rectangular opening with the help of a motorized manipulator. The small anchoring legs of the Microdrive were fixed onto the skull surface with dental cement (Tetric evoflow, Ivoclar Vivadent GmbH). A second small opening was made above the cerebellum to lead the silver ground wire between the skull and dura. This wire was anchored into the small hole conductive silver epoxy and dental cement.

The electrode tips were lowered into the brain with the help of a small screwdriver, while the Microdrive was still held by the manipulator. When all the tips penetrated the brain, we used a silicone gel (3–4680, Dow Corning, Midland, MI) as an artificial dural sealant for these long-term electrophysiological experiments. Dural sealants are important to preserve the integrity of the intracranial space after a craniotomy and in prolonging the lifetime and functionality of implanted brain probes. The upper part of the silver ground wire was fixed to the shuttle arm with dental cement, and we left only a smaller length of wire free, which could follow the movement of the shuttle on the Microdrive. The Microdrive was released from the motorized manipulator and the dummy weight was removed from the plastic backpack. The 16-channel amplifier board was attached to the plastic backpack and the Omnetics connectors of the MEA and amplifier board were coupled together. The birds received postoperative analgesia with Meloxicam (0.2–0.5 mg/kg, *im*) after they were released from the stereotaxic system.

After the surgical intervention, the birds were allowed to recover in a small cage (~30 min). All birds recovered quickly from the treatment, and we were able to release them back to their home cage into the sound box.

The surgical procedures for the anesthetized preparation were very similar although it did not require the use of a microdrive, instead a holding pin was cemented onto the skull to hold the head fixed during the recording session and the electrode arrays were lowered with a motorized micromanipulator. The anesthetized preparation involved two surgeries. Twenty-four hours prior to the neural recordings, the birds were deeply anesthetized with Isoflurane in order to cement the head post on the skull and to make fiducial marks to note the projected location of the electrode array penetration. On the day of the neural recordings, the birds were anesthetized using 3 injections of 25 μL of 20% Urethane administered at 30-min intervals. Additional details for the anesthetized procedure can be found in.^7^

#### Playback experiment and stimuli

Playback experiments for the awake experiments were performed according to the following protocol. On day 0, a male and a female zebra finch were put into the standard cage in the modified sound box for two weeks adaptation and pair bonding time. On day 10, the pair-bonded birds were equipped with a plastic backpack fixed with a rubber cord (Figure 1). The upper surface of the plastic backpack is covered with Velcro tape. Small dummy weights (~1g) were then attached onto these backpacks for further adaption to the recording setup. Once the birds were completely comfortable with the weights, these were replaced by the 16-channel amplifier boards (RHD2132 16-Input Amplifier Board, Intan Technologies) connected to the headstage (Figure 1). These amplifier boards filter, amplify, and digitize the neural signals recorded from the 16 electrodes. This recording set-up minimizes the weight on the bird’s head and maximizes mobility and comfort since the bird is tethered close to its center of mass; the birds can fly from perch to perch and exhibit a range of normal behaviors including vocal communication. Although in this preparation neural recordings can be obtained continuously, in these experiments we recorded 4–8 h during the day only. During these long recordings, the neural signal varied somewhat. Thus, we implemented a time-varying spike-sorting algorithm (see below) that allowed us, not only to determine when the activity of a particular unit became detectable and then again undetectable, but also to make sure that we keep track of any unit through time despite its spike shape variations.

The neuronal channels from the 16-channel amplifier board, the external USB microphone and the sound played back by the speaker were fed into the Intan RHD USB interface board. The audio data of the last two mentioned devices went through two separated custom-made I/O voltage level shifters. These are circuit boards to scale analogue voltages in the range of ±3.3V to the range of 0–3.3V for sensing by the RHD2000 interface board. We used a stereo HI-FI amplifier system (Dynavox CS-PA1 Mini Amplifier) between the ‘‘Line-out’’ jack of the desktop computer and the speaker inside the sound box.

Sounds were broadcasted in a pseudo random order and with inter-stimulus intervals with random uniform distribution between 3 and 6 s using a custom-written python code based on the pyoperant package written by the Gentner lab at UCSD (https://github.com/gentnerlab/pyoperant). The stimuli consisted of different calls and songs belonging to 10 of the ethogram-based call-types of the zebra finch repertoire. These stimuli were chosen from the larger dataset of calls and songs collected in the Theunissen lab from 3405 vocalization bouts in 45 zebra finches. A complete description of this database, the behavioral meaning of the call-types and their acoustic properties can be found in.^21^ A brief description is also found in the main text. For the awake dataset we used 110 distinct stimuli sampling 10 call-types (Begging or Be: 12; Long-Tonal or LT: 13; Thuk or Th: 5; Distress of Di: 5; Aggressive or Ag: 9; Whine or Wh: 13; Nest or Ne: 15; Tet or Te: 15; Distance Call or DC: 14; Song or So: 9). Although we always played-back 10 trials for each stimulus (for a total of 1100 playbacks) at each site, we did not always record 10 trials of data for each unit, either because of movement artifacts or because we lost the unit. We only analyzed data for which we had at least 2 trials to each of the 110 distinct stimuli. In the anesthetized experiments, we used 123 stimuli sampling 9 call-types (Be: 10; LT: 8; Th: 8; Di: 9; Ag: 11; Ne: 15; Te: 18; DC: 17; So: 27). Since we only played Whines (Wh) in one bird in the anesthetized dataset, we decided to not include those data in our analyses. When the anesthetized and awake data are compared (as in SI Figure S4), the analyses for the awake dataset were repeated also excluding Whine calls. In the anesthetized dataset, the actual number of stimuli obtained for each unit varied between 29 and 123 but we only analyzed data for which we had unit responses for 100 or more stimuli. In the large majority of cases, we obtained 10 trials for each unique sound (actual range: 8 to 20). The sound level during playback presentation was matched to that obtained during our recording of these calls 20 cm at top of their cage and varied for each stimulus mimicking the natural variation in loudness both across call-types and particular renditions (see also^7^ for additional details on sound stimuli).

#### Electrophysiology and spike sorting

For the awake preparation, one day after the operation, extracellular electrophysiological recordings were performed in the custom-made sound boxes, using the Intan hardware (RHD USB Interface Board, Intan Technologies) together with the USB Interface Board software. Before the recordings were started, birds were tethered by a thin cable (RHD2000 Ultra Thin SPI Interface Cable, Length: 0.9 m, Intan Technologies) that linked their amplifier board on their backs and to a high-quality mechanical commutator (Dragonfly Research and Development) placed on the top of their cage. The electrodes were lowered into the brain until we could identify auditory neurons. Later we used daily 0.5–1.0 turn (1t = 250 μm) steps further lowering the electrodes in the dorsoventral direction. Data were collected from the birds for 28–31 days until electrode tips reached the 10.0t - 11.0t depth. During the daily recording times the birds were separated inside their cage with a plastic wall, which consisted of a plastic frame and mesh. In this way the birds could see and hear each other during the recording times. The grounding cable of the custom-made Faraday cage inside the sound box was led out from the box and plugged into the input of ground for Faraday cage on the interface board. Neural responses were recorded using the signal of the 16 channel MEA with 25.0 kS/s amplifier sampling rate. Desired bandwidth was set to 170.00 Hz as lower and 10.00 kHz as upper bandwidth. After the end of the chronic recordings the position of the implanted MEA was verified. A custom-made lesion device was developed to have the possibility to do electrolytic lesions on selected electrode channels and at different depths. This device was connected to a linear stimulus isolator (WPI Inc.) and the output jumper cable was attached to the Omnetics connector of the MEA. A current of 100 μA was applied for 5 s. For the anesthetized preparation, details on the electrophysiological recordings can be found in.^7^

Electrode signals were high pass filtered at 300 Hz and the common average of all electrodes was subtracted from each channel. Putative spikes were identified as periods where the absolute value of the signal crossed a threshold of 4 times the standard deviation of the signal computed in 5-min blocks. These spikes were then sorted into units using a semi-automated procedure that includes an automatic step and a manual step.

To account for drift in spike shapes over time, the automatic step implements a custom hierarchical spike sorting procedure inspired by.^61^ First, the dimensionality of spike snippets is reduced using the non-linear embedding UMAP.^62^ Next, the spikes are over-clustered in short time windows of 2000 spikes using a Gaussian mixture model followed by cluster reassignment using ISO-SPLIT^63^ to split up multi-modal clusters. Next, a directed graph is formed by linking similar clusters from one time-window to the next (similarity measured using the Kolmogorov Smirnov test with spike shapes projected into 1D), and clusters with multiple incoming edges are further divided based on the distributions in nodes corresponding to those edges. Finally, this procedure is repeated for all spikes that were assigned to clusters that were too small to sort; this helps to identify spikes from low firing rate units after high firing rate clusters are identified in the first iteration.

In the manual step, a researcher manually splits up and joins spike clusters while deleting clusters of noise through our custom GUI tool (https://github.com/theunissenlab/suss-sorter) using information about spike shape distribution, inter-spike interval (ISI) distribution, and/or the stimulus triggered PSTH. Clusters connected in the directed graph procedure described in the previous paragraph are suggested to the user as initial groupings of clusters which can then be split up or reassigned manually based on the additional context provided by the program. The manual step allows users to group together clusters in time that are clearly originating from the same neural source.

After spike sorting, signal to noise ratios (SNR) were used to label units as potential candidates for single neuron activity. The spike *signal* was defined as the difference between the maximum and the minimum of the average time-varying spike snippet across all snippets obtained for that unit. The spike *noise* was estimated by the rms of each single snippet, then averaged over all snippets. The snippet length was 48 sample points or 2 ms, centered on the peak. We used an SNR >5 as a threshold for labeling a unit as a potential single unit. During the periods contaminated with motion artifact, that spike rate for the potential single unit clusters dropped precipitously to zero or to very low values. Using a simple threshold of 1 Hz allowed us to detect all the times windows without noise artifact for particular electrodes and units. In the awake behaving dataset, the average recording duration for a potential single unit was 2h46mins (range: 0h:16m to 6h:30m). The average gap duration was 6m:44s (range: 1s to 2h:26m). The average number of gaps per recording session was 10 (range: 0 to 39). The average percent of the time during a recording session with reliable spike sorting was 64% (range: 17%–100%)

Finally, to further quantify the potential contamination of potential units defined using the SNR threshold, we used two quality indices that compare the number of spikes that are found in a 2 ms refractory period to the number of spikes expected from a homogeneous Poisson distribution with similar firing rate (SI Figure S11). For, the first quality index, Q_isi_, the rate of the Poisson distribution is obtained by fitting the distribution of the interspike intervals with an exponential distribution using the data between 10 and 100 ms. The calculation of the second quality index, Q_aca_, uses the same algorithm as in the spike sorting software Kilosort (github. com/MouseLand/Kilosort). In that algorithm, the rate is estimated from the autocorrelation function in two separate windows: default values define one window from 10 to 50 ms and another from 250 to 500 ms. The maximum of those two values is used in the denominator of the quality index. The average and quartile values for these two quality indices and their corresponding p values are shown in Table 1. Those data show acceptable contamination levels for 50% of our data and significant decreases in spike counts in the 2ms refractory period relative to the Poisson assumption for 75% of the data. Since the Q indices were systematically higher for units with low overall firing rate but bursty responses to vocalizations, we preferred to use the SNR measure for thresholding potential single units. A threshold value of 5 led to some contamination as shown here. For this reason, we refer to these candidate single units simply as units, with the understanding that some of them might reflect the activity of two or more neurons with similar spike shape recorded from the same electrode simultaneously or in succession across the gaps during which the signal was corrupted by motion artifacts. Although perfect single unit isolation is critical to answer specific questions (e.g. microcircuitry), simulations have shown that perfect spike sorting might not be required to analyze ensemble.^64^

In the awake dataset, we recorded the activity of 730 spike-sorted units of which 292 were classified as auditory (40.1%). In the anesthetized dataset 607 out of a total of 1207 spike-sorted units (50%) were classified as auditory using the same criterion (SI Figure S2).

#### Histology

Thirty minutes after electrolytic lesioning, subjects of the awake preparation were euthanized by overdose of isoflurane and brains were removed from the skulls. They were quickly frozen on dry ice and then stored in a −80°C freezer until cryosectioning. Frozen brains were cut in 25 μm sections by a cryostat (Leica CM3050 S, Leica Biosystems) for fluorescent (DiI) histological tracking and later for Nissl staining. The lesion sites and fluorescent electrode tracts were confirmed with a fluorescent/light microscope (Leica DM 6000 B, Leica Biosystems). Fluorescent images were digitized using a high sensitivity monochrome digital camera (Leica DFC350 FX, Leica Biosystems) and for Nissl stained slices a digital color camera was used (Leica DFC480, Leica Biosystems). The position of the electrode tracks confirmed that our auditory recordings were in the auditory caudal pallium and more precisely in CM, NCM, and the various subdivisions of Field L: L1, L2a, L2b, L and L3. Using the position of each electrode as recovered from histological slides and the depth of the recording site, we assigned units as being in CMM, CLM, NCM, Field L or at the border region between L and NCM (see SI Figure S5). For the dataset obtained in the anesthetized preparation, see^7^ for distribution of units assigned to each area of the auditory pallium.

### QUANTIFICATION AND STATISTICAL ANALYSIS

#### Data analysis

##### Response strength and rate-based selectivity

The neural response in terms of its firing rate was characterized by the response strength (Z): *Z* score of the difference in number of spikes in the 500 ms after the onset of the stimuli and the number of spikes in the 500 ms before the onset of the stimuli. To calculate the *Z* score, both the mean and the standard deviation of the difference in firing rate were calculated across all trials and all stimuli for each unit. A value of response strength (z-scored rate difference) was then assigned to each trial. The rate-based selectivity index was calculated for ethogram-based call-types using the average response strength over all trials and exemplars of a given call-type, *z*_*CT*_, and the average of those averaged z-scores across all call-types, $\bar{Z_{CT}}$. The selectivity index was then calculated using the formula suggested by^65^:

$$SI=\frac{1-\frac{{\bar{Z_{CT}}}^{2}}{Z_{CT}{\;}^{2}}}{1-\frac{1}{n}}$$

where n is the number of stimulus classes; here n = 10 call-types. The SI equals 0 when all call-types have the same average response strength (and *z*_*CT*_ is thus equal to $\bar{Z_{CT}}$) and equals 1 when the average response strength *z*_*CT*_ is zero for all call-types except one (and in that case $\bar{Z_{CT}}-Z_{CTi}/n$ with i being the non-null call-type.)

##### PC analysis

To represent the time-varying response with a small number of parameters, we used a principal component decomposition. The average time-varying response was first estimated for each stimulus and each unit using a kernel density estimation with Gaussian kernels of 30 ms (Python: KernelDensity() from *sklearn.neighbors*). In this step, we excluded stimuli for which a particular unit had fewer than 5 trials. We applied the principal component analysis (Python: PCA() from *sklearn.decomposition*) to this response matrix. The PCA was performed separately for the awake and anesthetized datasets. The first 5 PCs explained ~90% of the variance as shown in SI Figure S7. We then used the weightings on the first 5 PCs to represent the time varying response in each single trial: the weightings for single trials were obtained by directly projecting the time vector of spikes obtained from the spike arrival times onto each of the PCs.

##### Naive Bayesian decoder and decoder-based selectivity (Entropy Selectivity)

A Naive Gaussian Bayesian decoder algorithm (Python: *GaussianNB* from *sklearn.naive_bayes*) was used to generate confusion matrices of predicted call-types vs actual call-types from the neural responses. The naive decoder assumes that for each call-type and unit the probability distribution of response parameters (here the response strength or one of the PC weightings) is normally distributed:

$$p\left(x_{i}|y\right)=\frac{1}{\sqrt{2\pi \sigma_{y}^{2}}}e^{-\frac{{\left(x_{i}-\mu_{y}\right)}^{2}}{2\sigma_{y}^{2}}}$$

where y is the call-type and x_i_, the value of one of the parameters, i, used to describe the neural response. Moreover, one assumes that the distributions of different neural response parameters are independent both within and across units. The posterior probability of a call-type given the neural response is then given by:

$$p\left(y|x_{i},x_{2},\ldots ,x_{n}\right)\infty P\left(y\right)\prod_{i=1}^{n}P\left(x_{i}|y\right)$$

For example, in the decoding of call-types obtained for two units based on rate only, the response vector (x_1_, x_2_) is given by (Z_1_, Z_2_); for two units based on rate and the 5 PC coefficients [T1, ..T5] the response vector would be (z_1_, T1_1_, T2_1_, T3_1_, T4_1_,T5_1_, z_2_, T1_2_, T2_2_, T3_2_, T4_2_, T5_2_). During the fitting process, the mean and the variance of the normal distribution for each neural response parameter is estimated. For example, for an ensemble of two units and a neural response based on response strength Z and each of the 5 PC coefficients describing the time varying response T, and 10 categories the decoder fit involves the estimation of 2 × 6 × 10 (for the means) + 2 × 6 × 10 (for the variance) = 120 + 120 = 240 parameters. For ensembles of 20 units, 2400 parameters will be fitted.

The decoder performance given by the full posterior probability was assessed by cross-validation. These posteriors were averaged across ensembles to generate the confusion matrices shown in Figure 4 and SI Figure S4. The percent correct classification (PCC) is obtained by the average of the diagonal of these confusion matrices.

In the cross-validation procedure, for each call-type, the responses to one of the exemplar stimuli was removed from the fitting procedure and used (as a testing set) to estimate the posterior. In this manner, we calculated decoder performance for increasing number of neurons and found that we reached a limit around 20 neurons. Larger ensembles led to overfitting and a decrease in decoding performance as the number of common stimuli played for all units in such larger ensembles started to decrease. Thus, the data limitation for these ensemble analyses is not due to the number of units recorded but instead on the number of stimuli per unit, which depended directly on the average recording time per unit.

To obtain the curve of decoder performance as a function of the number of neurons in the ensemble, we used a single ‘‘leave-one-out’’ per call-type for cross-validation. The estimation of the decoder performance for a single ensemble is in this case quite variable as it depends on that particular testing set. But we estimated the posterior probability for all combinations of ensembles of 1 and 2 units and for 10,000 randomly selected ensembles of size greater than 2 units. Since we calculated average performance using these 10,000 random samples, the fact that we used only a single ‘‘leave-one-out’’ for estimating decoder performance did not affect those average results (the standard error estimated for the sample of 100 neurons is too small to be visible on Figure 4B). To make sure we did not obtain a positively biased curve for the top 5% of these random 10,000 samples (dotted lines on Figure 4B), we re-estimated the value of the decoder performance for the top 5% obtained in the first round of decoding using a second round of fitting and testing with a second randomly chosen ‘‘leave-one-out’’ stimulus per call-type. These are the values used in the plots.

Finally, we also wanted to obtain better estimates of the model performances for ensembles of 20 neurons so that we could examine the relationship between ensemble performance and neural properties as shown in Figures 5E–5H. For this purpose, we used 100 leave one out, cross-validations for each ensemble. The number of ensembles was set at 1800 randomly chosen combinations of 20. However, the increase of cross-validation folds could only be performed for combinations of units that had minimum numbers of trials and stimuli. From the awake dataset, we selected units (n = 100) that had 110 stimuli and a unitary decoding performance PCC above 12% for the 10 call-types; chance is 10% and 2% is a rough estimate of SE for the Null hypothesis based on values obtained below 10% (see Figure 4). These *good* decoding units came from all 4 birds (ZF4F:43, ZF5M:19, ZF6M:16, ZF7F:22). From the anesthetized dataset, we selected units (n = 375) for which we had 100 stimuli for the 9 call-types and PCCs above 13% (chance is 11.1%). These good decoding units were also found in the 4 experimental birds (BlaBro09xxF:21, GreBlu9508M:171, Whi-Whi4522M: 83, YelBlu6903F:100). Ultimately, we obtained 1746 (sampled from n = 100 units) and 1348 (sampled from n = 375 units) ensembles of 20 units, for the awake and anesthetized dataset.

The confusion matrix obtained for units based on the response strength (Z) and time varying components (T) allowed us to estimate an alternative measure of neural selectivity that was based on the diagonal of this confusion matrix. Units for which the diagonal shows very good discrimination for one call-type but poor discriminations for all other call-types would be called highly selective, whereas units that show equal (average to poor) decoding performance for all-call-types would be called lowly selective. For this purpose, we estimated the normalized entropy obtained from the probabilities in the diagonals (PCC_c_ in the equations below) and defined an entropy-based selectivity measure (here called Entropy Selectivity) that we had called Global Selectivity (GS) previously in^7^:

$$GS=1-\frac{H_{obs}}{H_{max}}$$

where

$$H_{obs}=\sum_{c=1}^{10}-PCC_{c}\cdot {\text{log}}_{2}\left(PCC_{c}\right)$$

and

$$H_{\max}={\text{log}}_{2}\;\;10$$

Since the decoder took into account the temporal patterns in the neural response, the Entropy Selectivity also takes into account spike patterns, in contrast to the SI defined above.

#### Modulation receptive fields (MRFs) and acoustic space spanned by an ensemble of MRFs

The acoustic structure in our stimuli was described by the modulation power spectrum (MPS). The MPS were obtained from a 2D Fourier transform of the log amplitude of the spectrogram. The spectrogram was obtained by applying a short time Fourier transform (STFT) on the sound pressure waveform with Gaussian windows of bandwidth (in std) of 50 Hz or equivalently in the time domain 3.18 ms. This time-frequency scale sets the resolution of the spectrogram and upper limits of the MPS. The entire window in the STFT was 6 std or 19 ms and the window was shifted at 1 ms intervals. The MPS was obtained by windowing the spectrogram also with a Gaussian window of bandwidth (in std) of 16.7 ms for a total window size of 100 ms and a shift of 33 ms. This second window determines the number of time samples and with the upper limit determines the temporal resolution of the MPS. The spectral resolution was set by the upper frequency in the spectrogram set at 10 kHz. MPS were estimated for the first 500 ms of the stimulus since this was the window of analysis used throughout this study. MPS were calculated both for single stimuli and averaged across call-types. Before calculating the MPS, the amplitude in the spectrogram was normalized. In this manner the MPS only reflects the joint spectral/temporal structure in the sounds and not differences in sound level. The spectrograms and MPS were estimated using our own sound analysis Python package called BioSound (http://github.com/theunissenlab/BioSoundTutorial).

The modulation receptive fields (MRFs) were then obtained by performing a weighted average of the MPS obtained for each stimulus, where the weight was given by the neural response, either the response strength Z or the time varying components T. Thus, each neuron was characterized by 6 MRFs, one corresponding to the response strength and one for each of the 5 PCs used to describe the time-varying neural response. Before performing the weighted average, the MPS were zeroed by removing the mean MPS across all stimuli. The MPS were not however whiten by normalization by the auto-covariance matrix that one could obtain from all MPS for all stimuli used in each experiment. In this manner, the MRFs shown here are analogous to spike-triggered average (STA) stimuli but are not traditional forward filters that could be used to generate predictions of neural responses to arbitrary stimuli. One could generate these forward filters to obtain predictions of neural responses of units but this was not the goal of this ensemble analysis. The STA form of the receptive field made more sense in our study since we then projected our MRFs in the subspace spanned by the MPS of our stimuli given by the Fisher linear discriminant analysis (LDA). The LDA was calculated using custom code in Python notebooks (http://github.com/theunissenlab: GenerateMLFilters.ipynb) that estimated the eigenvectors of the between covariance matrix divided by the within covariance matrix of the average MPS across call-types (between) and the average of the covariance of the single MPS within a call-type (within).

Finally, we also estimated the volume spanned by an ensemble of MRFs in the acoustic subspace defined by the first three Linear Discriminant dimensions. The volume was calculated by taking the determinant of the covariance matrix obtained from the x,y,z coordinates of each of the MRFs in this 3D acoustic space. Thus, since we considered both the MRF_Z_s and the MRF_T_s for each unit, this 3x3 covariance matrix was calculated from 20x6 = 120 vectors when examining the volume spanned by ensembles of 20 units.

#### Adjusted Rand Index

To compare unsupervised clusters of ensemble neural responses, unsupervised clusters of the vocalizations based on their acoustic features, and ethogram-based call-types, we used the adjusted Rand index (ARI):

$$RI=\frac{a+b}{\frac{n}{2}}$$

Where, given a set of n elements S, and two groupings of S, X and Y: *a* is the number of pairs of elements in S that are in the **same** subset in X and in the **same** subset in Y and *b* is the number of pairs of elements in S that are in **different** subsets in X and **different** subsets in Y

Thus, the Rand index can be thought of as the fraction of pairs of elements which are either both in the same group or both in different groups. The Adjusted Rand index (ARI) is a form of the Rand index corrected by the values expected by chance^66^:

$$ARI=\frac{RI-RI_{expected}}{RI_{\max}-RI_{expected}}$$

For a random labeling, the ARI will have a value close to 0 and for identical groupings of two clusterings will be exactly 1. The ARI is also independent of the number of elements, n.

#### Acoustic feature space

For the unsupervised hierarchical clustering performed on the vocalizations and shown on Figure 6, we used the complete recordings of the 3405 vocalization bouts obtained from 45 zebra finches as described above in ‘‘*Playback experiment and Stimuli*’’. These vocalizations bouts were further segmented into 7920 calls (including song motifs and song intro notes) by detecting gaps in the amplitude envelope. These individual calls were then characterized using 20 predefined acoustic features describing the shape and magnitude of amplitude envelope (mean T, std T, skew T, kurt T, entropy T, rms, max Amp), the shape of the spectral envelope (mean S, std S, skew S, kurt S, entropy S, Q1, Q2, Q3) and pitch features (saliency, mean F0, max F0, min F0, cv F0). Detailed descriptions of these acoustical parameters can be found in.^21^ The parameters were estimated using the python package BioSound (http://github.com/theunissenlab/BioSoundTutorial).

#### *UMAP and* hierarchical *clustering of Auditory stimuli\*

We used the non-linear embedding called Uniform Manifold Approximation and Projection (UMAP) both for dimensionality reduction for visualization and as a non-linear processing step for the hierarchical clustering. We visualized the acoustic subspace occupied by the vocalizations using the first 2 UMAP dimensions (shown in Figure 6). The 20 UMAP dimensions were used to calculate distances between vocalizations in hierarchical clustering used to obtain acoustic labels: hierarchical agglomerative clustering (linkage() in *scipy.cluster.hierarchy*) was applied to the UMAP projection space to produce a dendrogram representing the Hierarchical structure of the acoustic repertoire (Figure 6A). Each cut in this dendrogram represents a unique grouping of the acoustic space. Each unique grouping from the cuts of the dendrogram are evaluated using the ARI measure described above. The highest ARI grouping is reported in the text. The unique grouping with exactly 9 categories was used as the acoustic category label for each of the call stimuli.

#### Hierarchical clustering of neural ensemble activity

For a given ensemble of neurons, neural activity was reduced to five time-varying components T and the response strength Z per neuron, as described above in the “*Naive Bayesian Decoder and decoder based selectivity”* section, and averaged across presentations of the same call stimuli. Hierarchical agglomerative clustering was then used to generate a dendrogram representing the agglomerative structure of these ensemble neural responses. Then, similar to the way that a maximal ARI is calculated for the acoustic space, all cuts of the neural dendrogram are quantified by calculating the ARI comparing the labeling of the neural activity in response to a given stimuli (labeled from the cut of the dendrogram), to the label of that stimuli given by either its ethogram-based call-type (*ARI*_*call*_) or its acoustic cluster (*ARI*_*acoustic*_).

#### Statistical analysis

Classical statistical tests were performed but with an important difference when analyzing the ensemble responses. In our analyses, we are sampling from n = 100 (awake) or n = 375 (anesthetized) neurons to generate random ensembles of neurons up to size k = 20. The number of combinations given by ‘n choose k’ is very high and one could always generate enough ensembles to be certain of our estimates of the parameters (e.g. the mean, the sd) describing the distribution of values for this particular set of 100 neurons or 375 neurons. (Note: the sample sizes (n_s_) are smaller for the analyses per bird (see Figure S4) or per region (see SI. Figure S6). We wanted however to estimate the statistical significance for independent ensembles (i.e. without any overlap in units). Thus, we estimated *ensemble* corrected estimates of the variance of our main effects (e.g. decoder performance), the standard errors (se) and the degrees of freedom (df). All reported p values rare based on these ensemble corrected measures of se and df.

If each unit contributes equally to the ensemble measures, the corrected unbiased variance is given by:

$$s_{c}^{2}=\frac{n_{s}}{\left(n_{s}-n_{e}\right)}s_{nc}^{2}$$

Where $s_{c}^{2}$ is the corrected sample variance, $s_{nc}^{2}$is the sample variance calculated as usual by dividing the sum of square deviations from the mean by the number of ensembles randomly generated minus 1, *n*_*s*_ is the sample size (e.g. 100) and *n*_*e*_ is the size of the ensemble (e.g. 20). The effective sample size is:

$$n_{eff}=n_{s}-n_{e}+1$$

from which one can estimate pooled variances, standard errors and obtain the corrected df for performing classical statistical pairwise tests (e.g. t-tests). For example, the standard error for a mean estimate will be obtained by:

$$sem=s_{c}/sqrt\left(n_{eff}\right)$$

In multiple linear regression analyses (including bivariate analyses), the standard $R_{adj}^{2}$ will give an unbiased effect size as both the mean square error and the variance, on the numerator and denominator would be corrected by the same factor to obtain corrected mean square errors:

$$R_{adj}^{2}=1-\frac{SS_{e}/\left(n-k\right)}{SS_{t}/\left(n-1\right)}$$

Here n is the total number of ensembles sampled in the sum of square errors (SS_e_) and sum of square deviations from the mean (SS_t_). For example, for 10,000 sampled ensembles, n = 10,000.

In the calculation of the F value, the sum of square errors in the numerator and denominator are also affected equally by the non-independence of the ensembles. In addition, the difference in sum of errors found in the numerator should be the one that is obtained with the effective sample size and not the number of ensembles sampled.

$$F=\frac{n_{eff}}{n}\frac{\left(SS_{t}-SS_{e}\right)/\left(k-1\right)}{s_{e}^{2}}$$

where

$$s_{e}^{2}=\frac{SS_{e}}{\left(n-k\right)},n_{eff}=n_{s}-n_{e}+1\left(\text{as defined above}\right)$$

and n is the number of ensembles sampled.

Finally, the F-test requires using the F-distribution with the denominator degrees of freedom given by:

$$df=n_{eff}-k$$

and the numerator degrees of freedom given by:

df=k−1

## Supplementary Material

1

2

## Figures and Tables

**Table 1.** Quality indices, Q_isi_ and Q_acc_, and the probability of obtaining such values P_isi_ and P_acc_ by chance based on a homogeneous Poisson distribution and the same number of total events

	SNR	Q_isi_	P_isi_	Q_acc_	P_acc_
Spike sort statistics for awake behaving dataset					

Mean	7.42	0.99	0.32	0.56	0.165
SD	2.52	1.65	0.45	0.47	0.346
25%	5.51	0.12	3.6e–52	0.16	0
50%	6.50	0.62	1.07e–5	0.52	9.40e–10
75%	9.03	1.11	0.99	0.84	0.0494

Spike sort statistics for anesthetized dataset					

Mean	6.56	1.35	0.37	0.51	0.098
SD	1.43	2.05	0.47	0.41	0.262
25%	5.57	0.37	1.65e–58	0.25	0
50%	6.21	0.74	1.33e–6	0.43	1.94e–16
75%	7.15	1.39	1.0	0.69	0.00102

**Table:** KEY RESOURCES TABLE

REAGENT or RESOURCE	SOURCE	IDENTIFIER
Chemicals, peptides, and recombinant proteins		

DiI powder	Thermo Fisher Scientific	D282, Invitrogen
Dental cement	Ivoclar Vivadent GmbH	Tetric evoflow
Medical Silicone	DOW Corning Corp., USA	Silastic Medical Adhesive Silicone Type A
Artificial dural sealant	Dow Silicones Corporation, Midland, MI	DOWSIL ™ 3–4680 Silicone Gel Kit

Deposited data		

Freely Behaving Neural Recordings and Stimuli.	This paper	https://doi.org/10.6080/K0TT4P5Q
Anesthetized Neural Recordings and Stimuli.	Elie and Theunissen^7,8^	https://doi.org/10.6080/K00C4T06

Experimental models: Organisms/strains		

Zebra Finch	Laboratory of Manfred Gahr, Seewiesen, MPI and of Frederic Theunissen, UC	N/A
Software and algorithms		
BioSound Code	Elie and Theunissen^20,21^	https://github.com/theunissenlab/soundsig (v1.0.1) https://doi.org/10.5281/zenodo.7378476
BioSound Tutorials	Elie and Theunissen^20,21^	https://github.com/theunissenlab/BioSoundTutorial (v1.0) https://doi.org/10.5281/zenodo.7378480
Spike Sorting Code	This paper	https://github.com/theunissenlab/suss-sorter (v0.2.1) https://doi.org/10.5281/zenodo.7378456
Analysis Code (Ensemble Decoding, MRFs calculations, statistics and figures)	This paper	https://github.com/ftheunissen/Ensemble-Code-Cell-Reports-2022 (v1.0) https://doi.org/10.5281/zenodo.7378502

Other		

Multi Electrode Arrays	Microprobe	Custom MEA-PI/Ir with Flexible Cable 16ch + Ref.
Intan Amplifier Board (Headstage)	Intan Technologies	RHD2132 16-Input Amplifier Board
USB microphone	audio-technica	AT2020USB+
Slip-ring miniature commutator	Dragonfly Inc.	SL-88-10
Ultrathin SPI Interface cable	Intan Technologies	RHD2000 ultrathin SPI Interface cable
SPI Cable Adapter Board	Intan Technologies	RHD2000 SPI Cable Adapter Board
USB interface board	Intan Technologies	RHD USB interface board (Part #C3100)
Speaker	VISATON	FRS 8, 30 w, 8 Ω
Desoldering copper wire	STANNOL GmbH & Co. KG	Stannol No-Clean Desoldering Wick, Product Nr.: 870,051

## Highlights

- Avian auditory cortical neurons categorize communication sounds
- This auditory categorization requires an ensemble neural code
- Neurons in the ensemble do not have to be selective for individual call types
- Neural categories match ethologically defined call categories above acoustics
