# Supplementary material for: Sparse ensemble neural code for a complete vocal repertoire
Source: Cell Rep. Author manuscript; Available in PMC 2023 Oct 23. (PMC10363576; doi:10.1016/j.celrep.2023.112034)
Supplement: 1 [file NIHMS1878912-supplement-1.pdf]

Cell Reports, Volume *42*

Supplemental information

Sparse ensemble neural code for a complete vocal repertoire

H. Robotka, L. Thomas, K. Yu, W. Wood, J.E. Elie, M. Gahr, and F.E. Theunissen

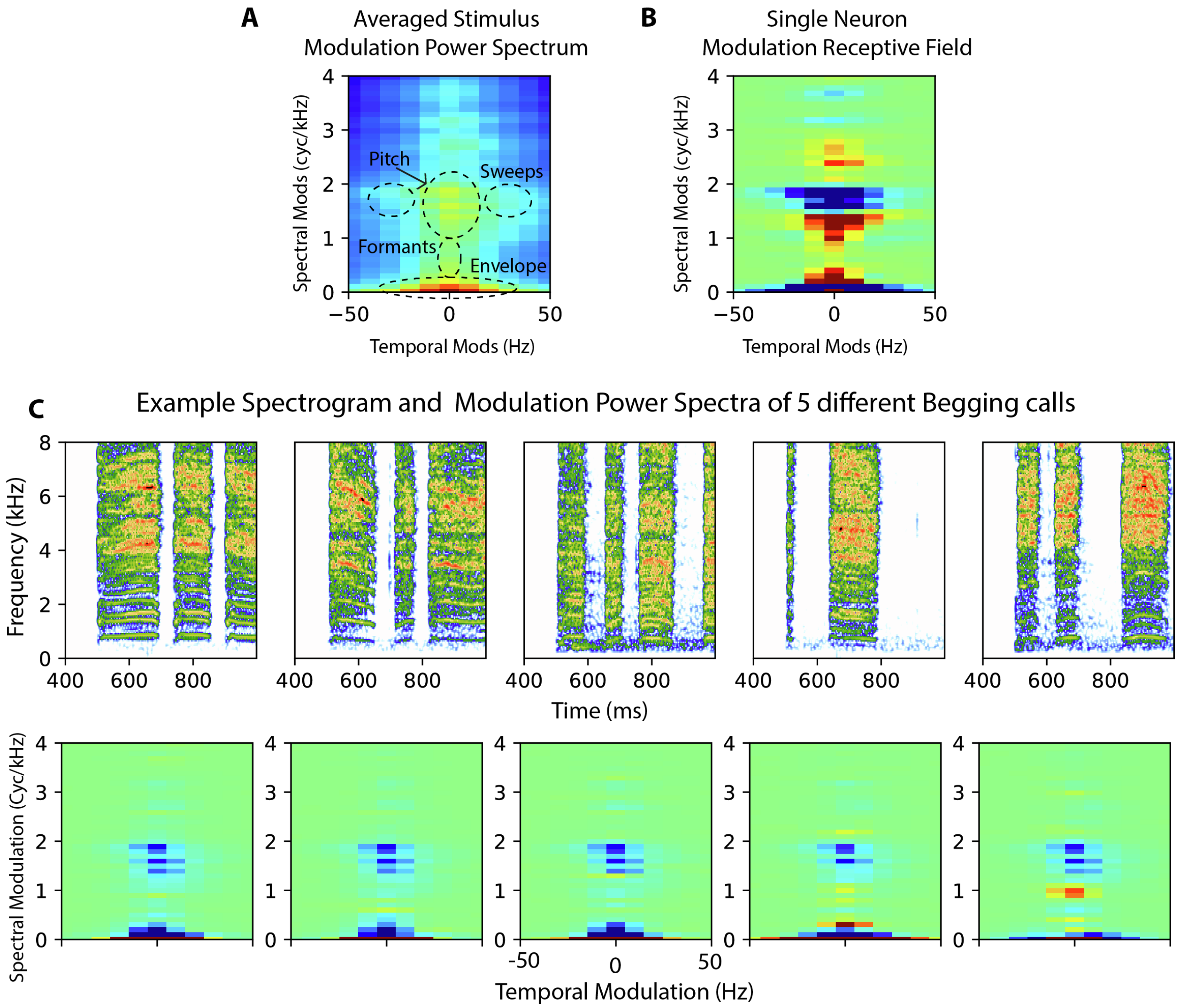

SI Fig 1. Modulation Power Spectra (MPS) and Modulation Receptive Field (MRF). Related to Figure 2 and 5
A. The average modulation power spectrum (MPS) of the 110 communication calls used as stimuli in the awake neural recordings is shown in a color plot. The x-axis measures the modulations in the temporal envelope and the y-axis measures the modulations in the spectral envelope. The area of energy around 0 Hz and between 1 and 2 cyc/kHz (Pitch) reflects the high pitch-saliency (tonal) harmonic stacks in the vocal repertoire of the zebra finch with fundamentals of 1000 to 500 Hz. Up-sweeps and down-sweeps such as those observed in the male distance call are found to the left and the right (Sweeps). Calls are also distinguishable by different frequencies in their formants (Formants) and in the shape of their temporal envelope (Envelope). **B.** Each component of the modulation receptive field (MRF) of a single neuron is estimated by a weighted average of the stimulus MPS deviation from the mean MPS (shown in A). The deviation is estimated for each stimulus and weighted by the neuron's response, here its response strength Z. This example neuron has tuning for vocalizations that are tonal (i.e. with harmonic stacks) but with preference for higher pitch (closer to 1000 Hz than 500 Hz), strong formant modulation, and temporal envelope dominated by lower modulation frequencies: LT calls would be effective stimuli (see Fig 2 in the main paper). For examples of MRF components obtained with the 5 PC coefficients T of the time varying response see SI Fig 7. **C.** Spectrogram and modulation power spectrum for 5 distinct Begging calls (Be) used as stimuli**;** the MPS of each exemplar of Be varies somewhat but much less so than the corresponding spectrograms. The individual MPS have the characteristic signature observed in the mean MPS for Be shown in Fig 2 of the main paper.

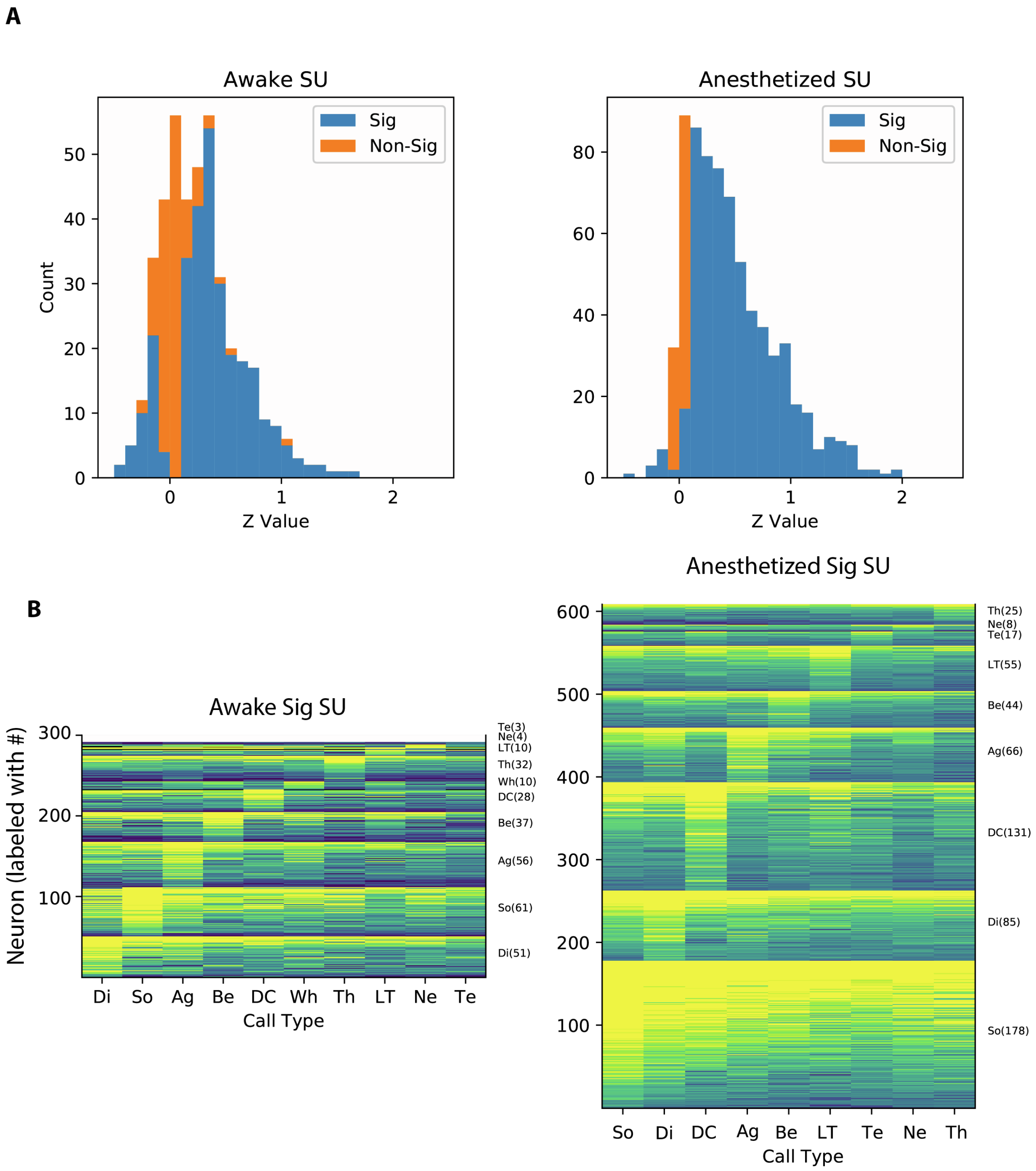

SI Fig 2. A. Stacked histogram of Auditory z-scores. Related to Figure 3. A z-score statistic is used to quantify the strength and sign of auditory responses based on the average stimulus evoked firing rate in a 500 ms window post stimulus onset relative to background rate in a 500 ms window preceding the stimulus onset. In the awake dataset (4 birds), 292 units had z scores that were significantly different from zero and 46/292 (15.7 %) that were inhibited by sounds. In the anesthetized dataset (here 6 birds but data from 4 are used in the decoding analyses), 609 units had auditory responses and 13/609 (2.1 %) were inhibited by sound. The range of z-scores was similar in both datasets. Significance is based on a two-tailed one-sample t-test with p-value<0.01. **B**. **Neural Responses to Repertoire.** As in main text Fig. 3C, each row of this color matrix shows the average response strength (Z) obtained in a unit for each ethogram-based call-type of the zebra finch repertoire. The left panel shows the neural responses for the awake dataset as in Fig. 3C (n=292). The right panel shows the neural responses for the anesthetized dataset for comparison (n=609). Note that the anesthetized dataset does not include the Whine call (Wh). See the legend of Fig. 3 for a description on how the data is organized on the plot.

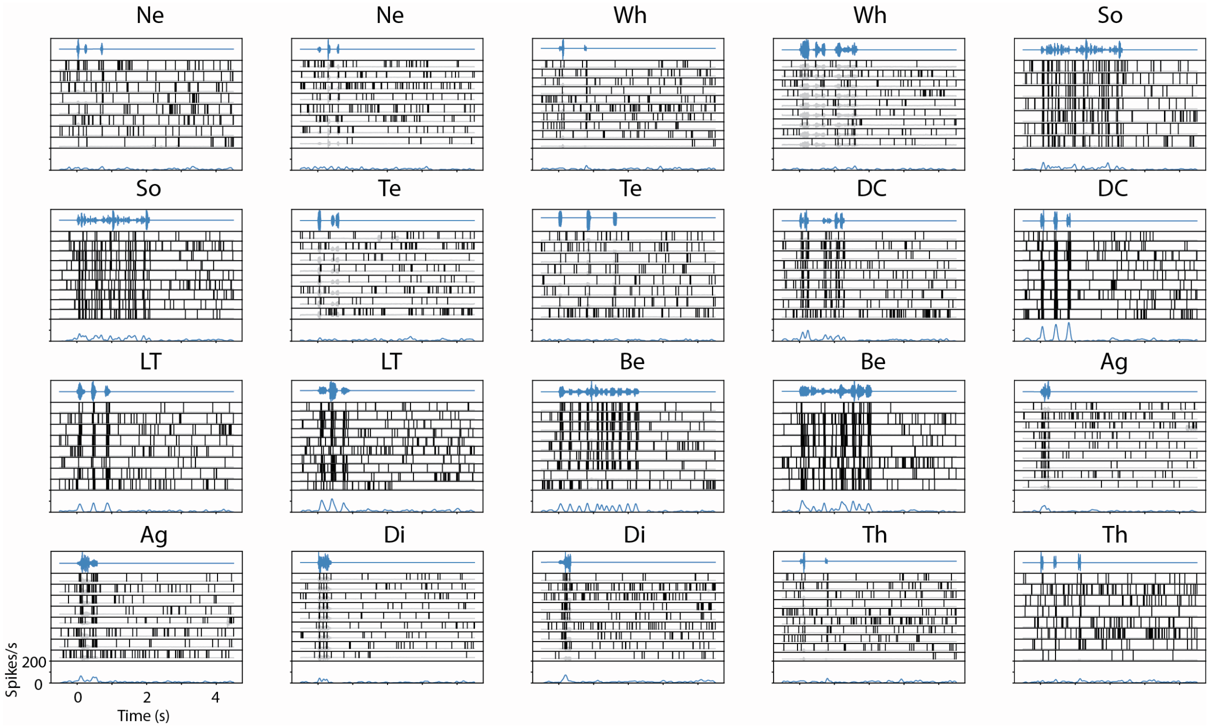

SI Fig. 3. Spike rasters obtained in response to the playback of example vocalizations (two from each call-type) for the intermediate selectivity unit shown in Fig 3A. For each example call, the top blue line is the oscillogram of the sound that was played back. The light grey lines depicted under the spikes (vertical black lines) of each trial row correspond to the ambient microphone trace (other sounds than the playback can appear in these traces). The bottom blue line is the smoothed (30 ms window) PSTH. The number of trials for each call was different for each unit but selectivity and decoding estimation were based on responses obtained for all 110 calls (only 20 calls are shown here).

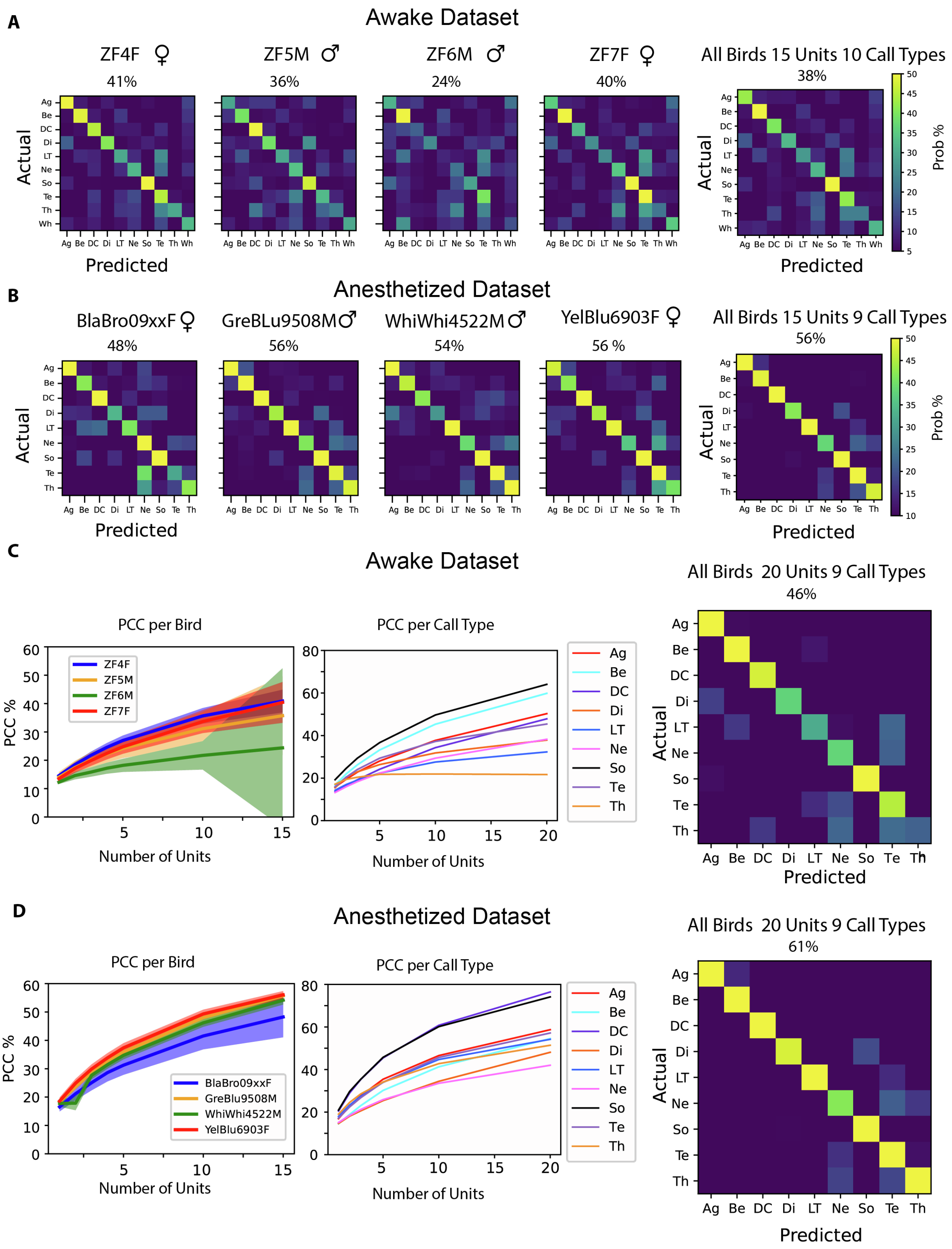
A
Awake Dataset
ZF4F ♀
41%
ZF5M ♂
36%
ZF6M ♂
24%
ZF7F ♀
40%
All Birds 15 Units 10 Call Types
38%
Actual
Predicted
Ag Be DC Di LT Ne So Te Th Wh
Prob %
50 45 40 35 30 25 20 15 10 5
B
Anesthetized Dataset
BlaBro09xxF ♀
48%
GreBLu9508M ♂
56%
WhiWhi4522M ♂
54%
YelBlu6903F ♀
56 %
All Birds 15 Units 9 Call Types
56%
Actual
Predicted
Ag Be DC Di LT Ne So Te Th
Prob %
50 45 40 35 30 25 20 15 10
C
Awake Dataset
PCC per Bird
ZF4F
ZF5M
ZF6M
ZF7F
PCC %
Number of Units
PCC per Call Type
Ag
Be
DC
Di
LT
Ne
So
Te
Th
Number of Units
All Birds 20 Units 9 Call Types
46%
Actual
Predicted
D
Anesthetized Dataset
PCC per Bird
BlaBro09xxF
GreBlu9508M
WhiWhi4522M
YelBlu6903F
PCC %
Number of Units
PCC per Call Type
Ag
Be
DC
Di
LT
Ne
So
Te
Th
Number of Units
All Birds 20 Units 9 Call Types
61%
Actual
Predicted

SI Fig 4. Decoder Performance per bird and per call-type. Related to Fig 4. A & B. In both the awake dataset and the anesthetized dataset, the ensemble decoding analyses were performed by randomly sampling ensemble of neurons of increasing size from data obtained in 4 birds for each dataset. Here we repeated this decoding analysis by only sampling from one bird at a time. We show the average confusion matrices obtained for each bird sub-dataset for ensembles of 15 neurons, in A for the awake dataset and all 10 call-types, and in B for the anesthetized dataset and 9 call-types (Wh call is missing). For comparison, the confusion matrices obtained using all birds data for ensembles of 15 neurons are shown on the most right column. As shown in both individual bird and lumped birds confusion matrices and in the average percent correct classification (PCC) per units in panels C and D, similar results are obtained in all birds both in terms of average performance (the ribbon error bars show two SE) and in terms of the systematic errors that can be seen in the confusion matrices. For example, the Thuk and the Nest calls are often misclassified as Tet calls. The corrected standard errors are large for ZF6M because of the small sample size for that bird. As described in more detail in the Methods, the numbers of units used in the decoder for each bird were: for the awake dataset ZF4F:43, ZF5M:19, ZF6M:16, ZF7F:22; and for the anesthetized dataset BlaBro09xxF:21, GreBlu9508M:171, WhiWhi4522M: 83, YelBlu6903F:100. For each of the awake and anesthetized dataset, we recorded the activity in two male birds and two female birds. We did not measure any significant differences in PCC between sexes (Awake: Diff PCC Female - Male = 8.3%, $t_{(2)}$= 0.6757 p= 0.5689; Anesthetized: Diff PCC Female – Male = 0.2%, $t_{(2)}$= 0.0268, p= 0.981) **C & D**. The line plots show the average PCC as a function of the number of units in neuronal ensembles sampled in each bird (left plots) and for each call-type center plots. The confusion matrices on the right are obtained for ensembles of 20 units in the awake and anesthetized dataset and both for 9 call-types (without the Wh in the awake dataset) so that the direct comparison can be made. The decoder performance is similar but higher in the anesthetized dataset. This increase in performance under anesthesia can be explained by a greater signal to noise ratio (SNR) in the neural response. This higher SNR is made evident in the average time varying rate that shows lower background rates relative to peak rates (compare black line on panels A and C in SI Fig. 7) and lower neural noise during the response (compare Fig 5H with SI Fig. 8H) in the anesthetized dataset. Note also that the systematic errors overlap not only across birds overlap but also across the two datasets.

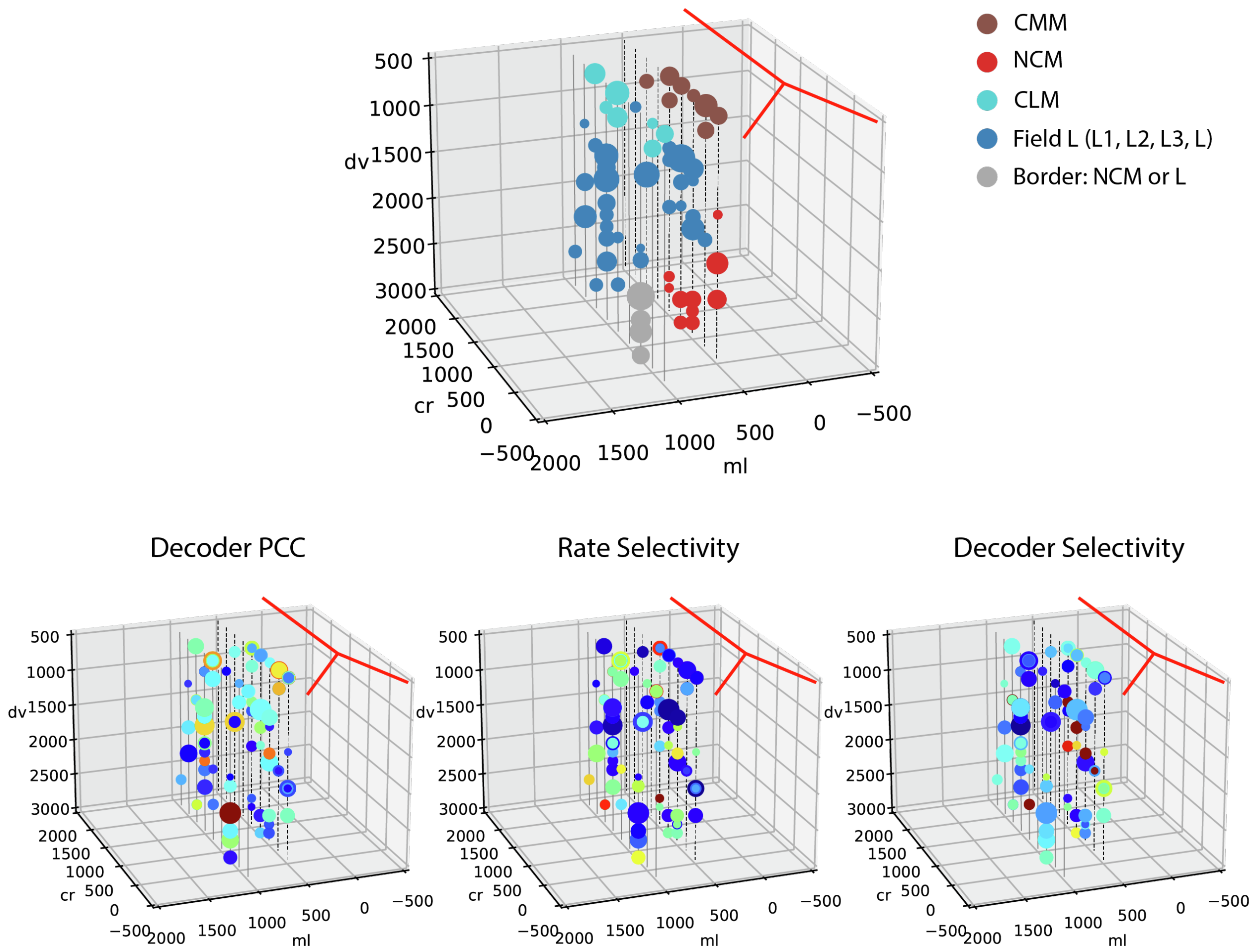

SI Fig. 5 Anatomical Location of Auditory Units and Response Properties. Related to Star Methods and Fig. 3. *Top Panel.* Using the histological reconstruction of electrode tracks (shown as dotted and grey vertical lines) and the depth of the recording sites, the 100 auditory and call-type discriminative units used in the analysis were assigned to different regions of the primary and secondary auditory pallium: 16 in CLM, 25 in CMM, 40 in Field L (L1, L2a, L2b, L, L3) and 13 in NCM. Six units were found in a region that is at the border between NCM and area L in Field L. For the ensemble decoding and tuning analyses the units were grouped into the 56 belonging to the primary auditory pallium (Field L/CLM complex) and the 38 belonging to the secondary auditory pallium (NCM/CMM). Units that were found at the border of NCM and the region L in Field L were excluded from these analyses. The anatomical location of the units is coded in the color of the spheres as shown in the legend. The size of the spheres scales with the standard deviation of the response rate calculated across call-types. The red line is a cartoon drawing of the approximate location of the y-sinus. A 3D movie version of this plot is available on-line. *Bottom Panels.* Same graphical representation as the top panel but where the color of the spheres correlates with the single auditory unit decoder performance in PCC (blue to red color scale: 10-20%), the Z-based Selectivity Index (SI, blue to red color scale: 0-1.0) and the decoder selectivity (Entropy Selectivity, blue to red color scale: 0-0.15). One can observe a great amount of local heterogeneity in these response properties and only weak effects across areas are observed. An ANOVA with anatomical region as the predictor suggested small differences across regions in decoder performance (R_{adj}^2= 0.059, $F_{(4,95)}$ = 2.558, p=0.0436), mean rate across regions (R_{adj}^2 = 0.084, $F_{(4,95)}$ = 2.7, p = 0.0351) and in Entropy Selectivity (R_{adj}^2 = 0.09, $F_{(4, 95)}$ = 3.54, p= 0.00965) but not SI (R_{adj}^2 = 0.038, $F_{(4,95)}$ = 1.9, p=0.102). Note that with the Bonferroni correction for the four statistical tests, the only classically significant result at the p=0.05 level is for the Entropy Selectivity. Z-based SI was highest in CLM and lowest in Field L and in the border area L (of Field L) and NCM with intermediate values in CMM and NCM. The decoder selectivity (Entropy Selectivity) which also takes into account the temporal pattern in the response followed the exact opposite trend: it was highest in Field L and lowest in CLM and CMM with intermediate values in NCM.

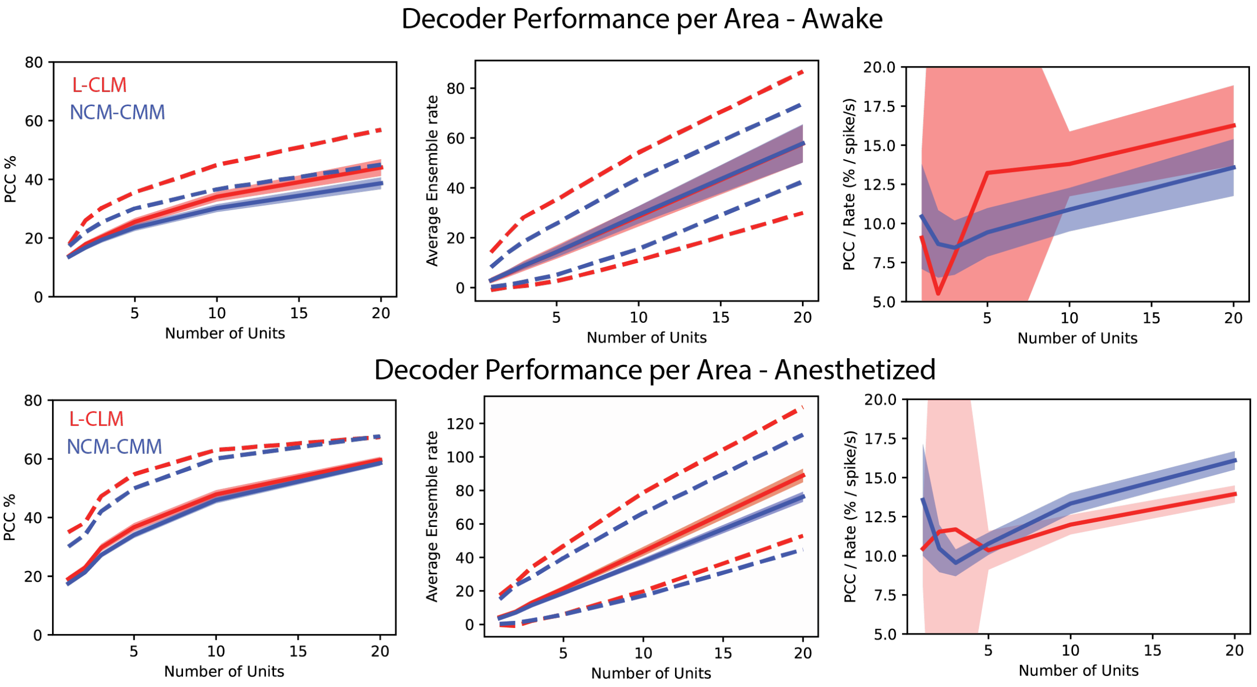

SI Fig 6. Decoder Performance per Area. Related to Fig. 4 and SI Figs. 4 and 5. The ensemble decoding analyses were performed after assigning auditory units to the primary auditory pallium, the Field L/CLM complex (labelled L-CLM in the figure) versus the secondary auditory pallium, NCM and CLM. In the awake dataset, 56 units were assigned to the primary auditory pallium and 38 to the secondary auditory pallium. In the anesthetized dataset, 209 units were assigned to the primary auditory pallium and 166 to the secondary auditory pallium. *Left Panels:* The ensemble decoder performance as a function of ensemble size was very similar in primary and secondary auditory areas. For ensembles of size 20, the measured differences were small and statistically significant at the 5% level in the awake dataset (d= 0.72, $t_{(54)}$= 2.55, p= 0.013) but not the anesthetized dataset (d= 0.186, $t_{(303)}$= 1.6, p= 0.11). Solid line and ribbon show average and 2 SEM. Dashed line shows average of top 5%. *Middle Panels:* The average response rates across call-types and summed for all units in the ensemble are plotted as a function of the number of units in the ensemble. These curves draw a linear function as expected. In the awake dataset, we did not observe differences in response rate of ensembles of 20 between the primary and secondary auditory pallium (Rate d= -0.006, $t_{(54)}$= -0.026, p= 0.98). In the anesthetized dataset, the response rate is higher in primary pallium (Rate d= 0.64, $t_{(303)}$= 5.53, $p<10^{-5}$). Solid line and ribbon show average and 2 SEM. Dashed lines show average of top 5% (top lines) and bottom 5% (bottom lines). *Right Panels:* To quantify the efficiency of the neural code, the decoder performance (PCC) of each ensemble was divided by the average rate of the units in that ensemble. This efficiency measure increases as the sample size increases, showing that a spike of a given unit is more informative when other units are considered. In the awake dataset, the coding efficiency in primary and secondary areas were not statistically different (d= 0.51, $t_{(54)}$= 1.81, p= 0.076). In the anesthetized dataset, the decoding performances are similar but the rate is lower in the secondary auditory pallium, yielding a higher efficiency of neural representation in the secondary auditory areas (PCC Norm d= -0.638, $t_{(303)}$= -5.514, $p<10^{-5}$). Solid line and ribbon show average and 2 SEM.

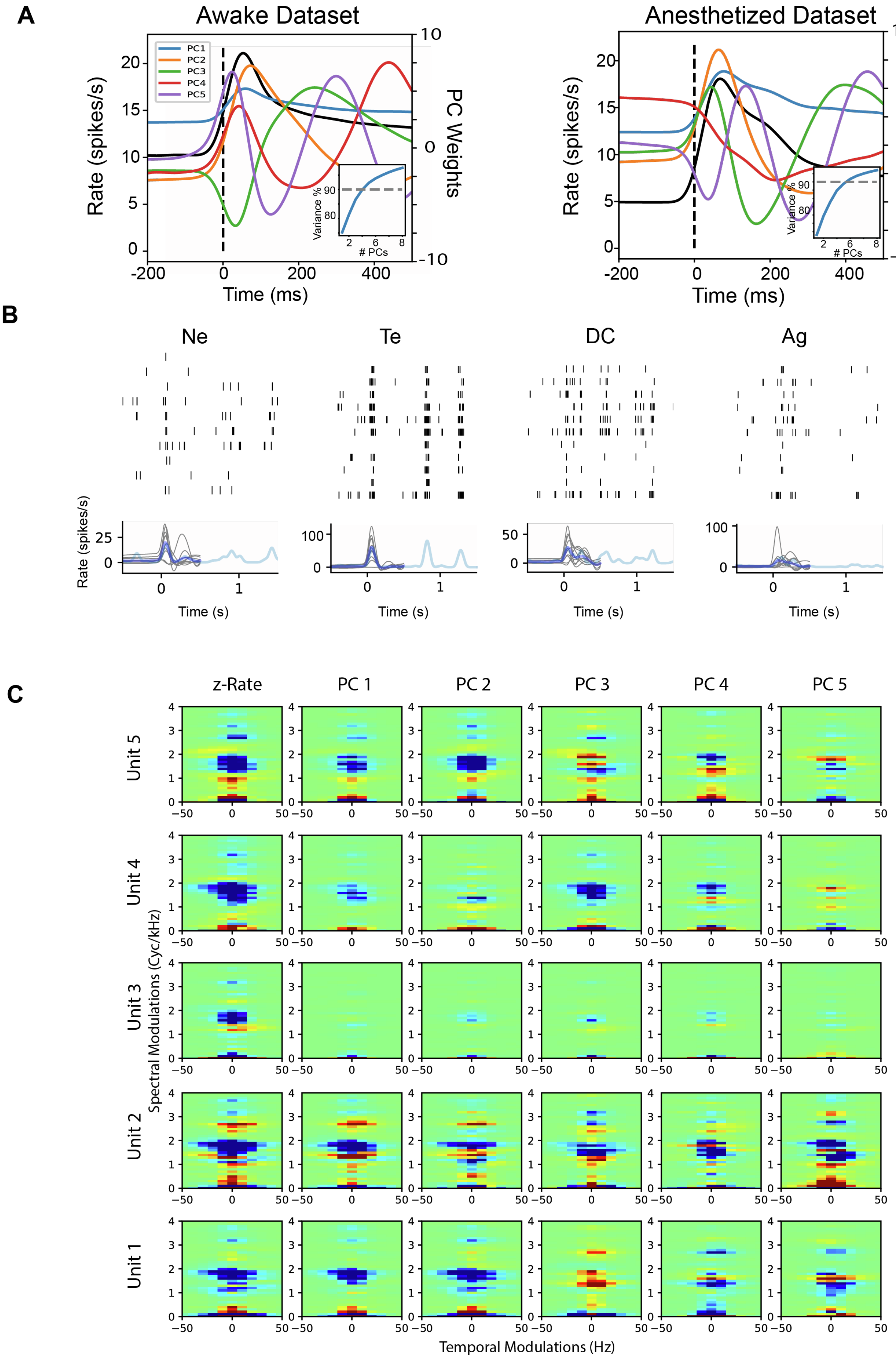
A
Awake Dataset
Anesthetized Dataset
PC1
PC2
PC3
PC4
PC5
Rate (spikes/s)
PC Weights
Time (ms)
Variance %
PCs
B
Ne
Te
DC
Ag
Rate (spikes/s)
Time (s)
C
z-Rate
PC 1
PC 2
PC 3
PC 4
PC 5
Unit 5
Unit 4
Unit 3
Unit 2
Unit 1
Spectral Modulations (Cyc/kHz)
Temporal Modulations (Hz)

SI Fig. 7. A. Principal Components of Time-varying Neural Responses. Related to Fig. 5. To capture the temporal patterns in the neural response spike trains with a minimum number of dimensions, we performed a principal component analysis on the time-varying trial-average responses to each vocalization from all units. The left panel shows the results of this analysis for the awake dataset (n= 292 units) and the right panel for the anesthetized dataset (n=609 units). The mean rate is shown as a black line with a scale on the left y-axis and the 5 first PCs are shown as colored lines with a right y-axis scale. Stimulus onset is at 0 ms and is shown with a dashed line. Insets show the cumulative variance explained as a function of the number of PCs: 5 PCs capture approximately 90% of the variance for both datasets. The weights of the PCs in the pre-stimulus period are constant, as expected, but can be negative or positive. These non-zero weights on the background rate reflect the fact that particular temporal patterns observed during peristimulus period are correlated with background rates that are below or above the average found across all neurons. Similarly, for any particular PC, there is a DC offset on its weights post stimulus onset. **B. Example Spike Rasters and Estimates of Time-varying Responses Obtained from PCs.** The upper plots show the spike rasters obtained from one auditory unit in response to the repeated presentations (rows) of one particular rendition of Ne, Te, DC and Ag each (columns). The bottom plots show the estimation of the time-varying rates. The light blue is the average time-varying rate obtained from the kernel density estimation applied to all spike trains obtained for each call-type rendition. The dark blue is the average time-varying rate reconstructed by using the 5 PCs shown in A. The two curves overlap almost perfectly as expected. The grey lines show the time-varying rate estimated for each repeated presentation also using the 5 PCs. It is these individual time-varying rates (represented by 5 numbers) that are used in the decoder (one set of 5 numbers per auditory unit and per trial) **C. Example of Modulation Receptive Fields (MRFs).** Each row depicts the 6 MRFs components obtained for an example unit from the awake dataset. The first component (MRF-Z, first column) is obtained from the neuron response strength (Z) to stimuli. The five other components (MRF-Ts, columns 2 to 6) are obtained from the five PC coefficients of the time varying response [T_1 T_2 … T_5]. The units were chosen to illustrate the heterogeneity in the neural tuning not only in terms of rate but also in terms of the information that can be extracted from the temporal pattern in the response. In Unit 1, MRF-Z, MRF-T_1 and MRF-T_2 will yield larger values for low pitch-saliency calls (noisy calls), while the MRF-T_3 will yield higher values for high pitch-saliency (tonal calls). The PC1 (blue) corresponding to MRF-T_1 predicts (mostly) the sustained activity while PC2 (orange) corresponding to MRF-T_2 predicts a transient response. Thus, a noisy call will trigger in Unit1 through MRF-T_1 and MRF-T_2 both an onset and sustained response. PC3 (green) can be used to create additional contrast between a late sustained response (positive weight around 250 ms) and the onset response (negative weight around 20 ms). For the same Unit 1, a noisy call will generate negative weights on PC3 via MRF-T_3, resulting in a greater than expected onset response relative to the late sustained, beyond what was already predicted by MRF-T1, MRF-T2. Unit 2 and Unit 4, show that MRF_Z and MRF_{T3} are not necessarily opposite in sign. These are units where the late sustained response will dominate. Unit 3 shows only a Z component MRF; variations in the temporal pattern do not provide additional information. In Unit 5, the spectral modulations that correspond to the pitch energy are well sampled, with visible increase in sensitivity for lower pitch (higher spectral modulations) from MRF_{T2} to MRF_{T4} to MRF_{T5}. The MRF_{Ts} may also vary significantly in the sensitivity for temporal modulations, for example the MRF_{T5} of Unit 2 shows high weights for fast amplitude modulations which is not observed in the other component MRFs of this unit.

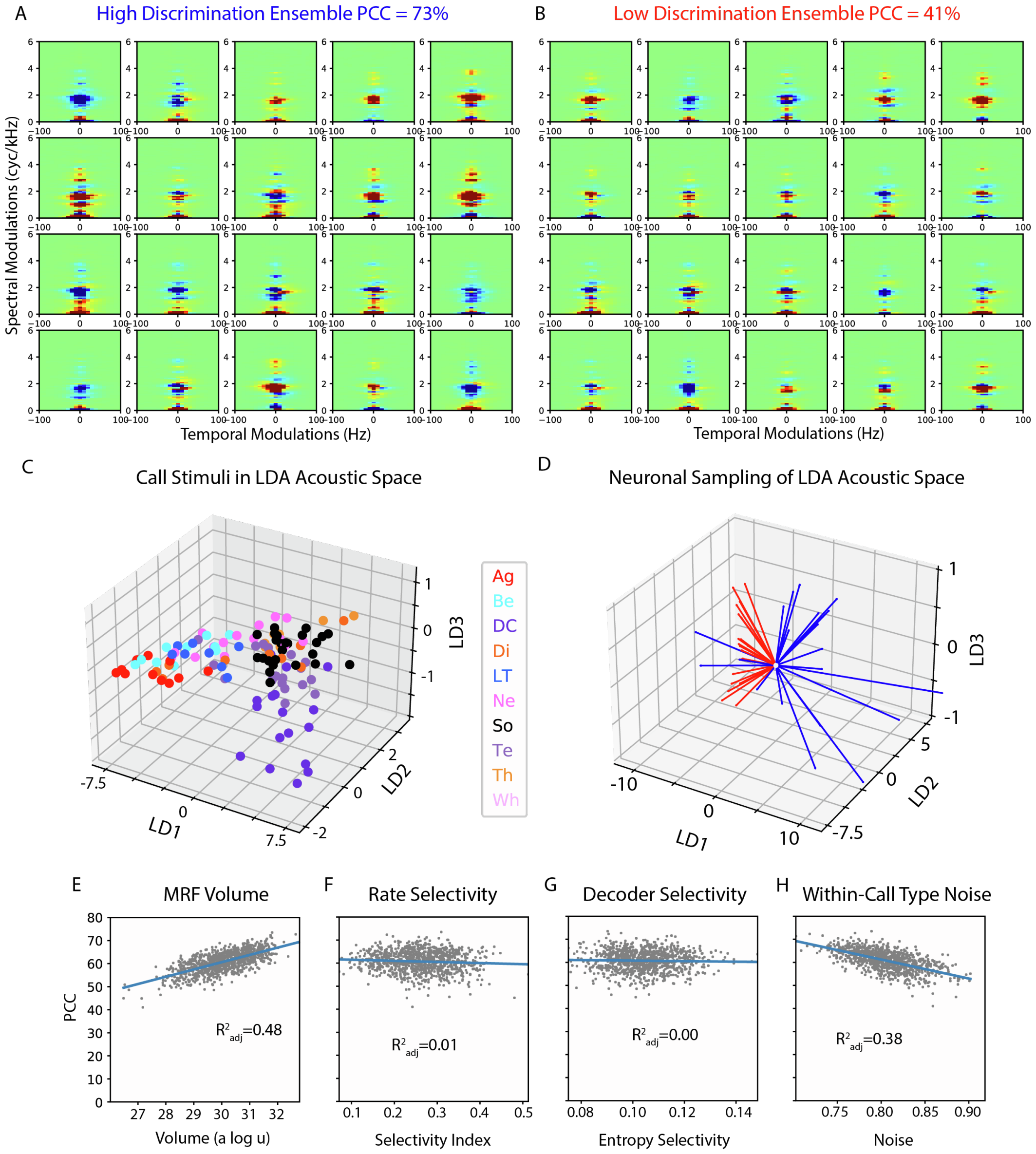

SI Fig 8. Ensemble Neural Tuning and Decoder Performance for the Anesthetized dataset. Related to Fig. 5. This figure is identical to Fig. 5 of the main paper but for the analyses performed on the anesthetized dataset. **A & B**. Modulation Receptive Fields based on response strength (MRF_Z) from a highly discriminating ensemble (A) and a low discriminating ensemble (B) each composed of 20 units. **C.** Projection of the modulation power spectrum (MPS) of the stimuli used in this experiment in the 3D space spanned by the first three linear discriminant functions (LDs). Note that the stimuli (chosen renditions for each call-type) used in the anesthetized dataset were different than the ones used in the awake dataset, although there was some overlap. The points shown here as well as the actual LD dimensions are different than those shown in Fig 5. Nonetheless, one can observe a similar parsing of the acoustic space along call-type categories. **D.** The MRF_Z shown in A and B projected as vectors in the same linear discriminant space as in C: the 20 blue vectors correspond to the 20 MRF-Z shown in A for the highly discriminating ensemble and the 20 red vectors correspond to the 20 MRF-Z in B. **E-F.** Bivariate regression analyses for random ensembles of 20 neurons (1,358 ensembles sampled from n=375 units) to analyze the relationship between tuning properties of the ensemble and the discrimination performance measured by the percentage of correct classification (PCC) (See Fig 5 for details). The coefficient of determination (R^2_{adj}) is significantly different from zero for E ($F_{(1,343)}$ = 314.8, $p<10^{-5}$) and H ($F_{(1, 343)}$ = 209.5, corrected $p< 10^{-5}$).

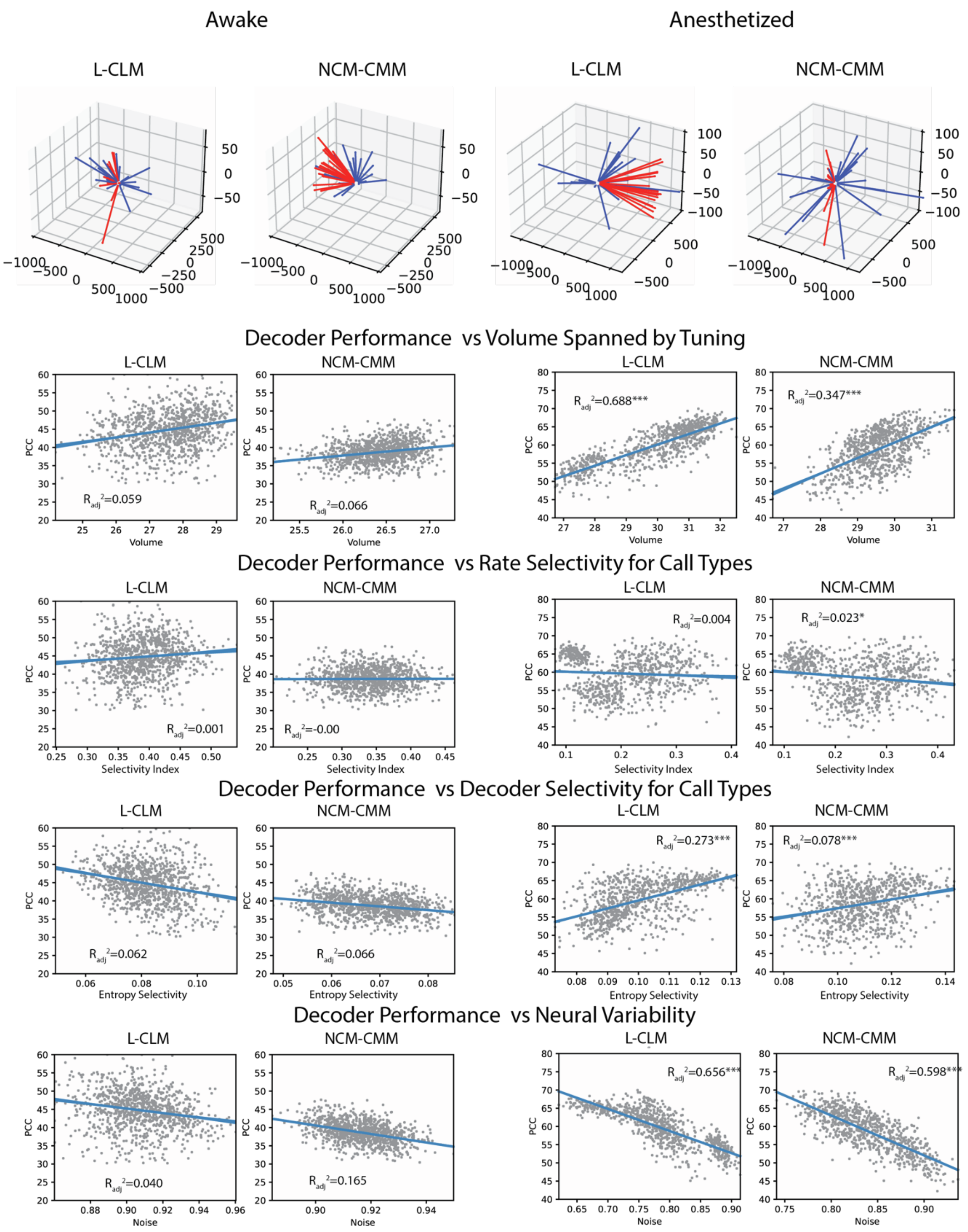

Awake
Anesthetized
L-CLM
NCM-CMM
L-CLM
NCM-CMM
Decoder Performance vs Volume Spanned by Tuning
L-CLM
NCM-CMM
L-CLM
NCM-CMM
R_adj^2=0.059
R_adj^2=0.066
R_adj^2=0.688***
R_adj^2=0.347***
PCC
Volume
Decoder Performance vs Rate Selectivity for Call Types
L-CLM
NCM-CMM
L-CLM
NCM-CMM
R_adj^2=0.001
R_adj^2=-0.00
R_adj^2=0.004
R_adj^2=0.023*
Selectivity Index
Decoder Performance vs Decoder Selectivity for Call Types
L-CLM
NCM-CMM
L-CLM
NCM-CMM
R_adj^2=0.062
R_adj^2=0.066
R_adj^2=0.273***
R_adj^2=0.078***
Entropy Selectivity
Decoder Performance vs Neural Variability
L-CLM
NCM-CMM
L-CLM
NCM-CMM
R_adj^2=0.040
R_adj^2=0.165
R_adj^2=0.656***
R_adj^2=0.598***
Noise

SI Fig 9. Ensemble Neural Tuning and Decoding Performance Analyzed per Area. Related to Fig. 5 As in Fig. 5 and SI Fig. 8, the relationship between the decoder performance and the tuning and response properties of units in the ensemble are analyzed by separating the units assigned to the primary auditory pallium (L-CLM) from those assigned to the secondary auditory Pallium (NCM-CMM). As illustrated in the top row, the tuning vectors of the best ensemble in each group (shown in blue) span a larger volume of the acoustic space occupied by call-types than the worst ensemble in each group (shown in red). The relationship between decoder performance and volume spanned was observed in both the primary and secondary auditory pallium and in both the awake and anesthetized sets but was statistically significant only in the anesthetized dataset for which we had a larger sample size and less variability. The effect sizes were much larger in the anesthetized dataset potentially because the total variance observed was closer to the explainable variance; the empirically measured neural variability was smaller in the anesthetized dataset. In the anesthetized dataset, the effect was greater in primary auditory area than in the secondary auditory area, suggesting that the MRFs were better descriptors of the stimulus response function of the neurons. The decoder performance was also negatively correlated with the neural variability in both the primary and secondary auditory pallium. The trend is clear both in the awake and anesthetized datasets but reached statistical significance only in the anesthetized dataset. Note the higher noise range in the awake dataset. In general, average Z-based selectivity (SI) of the units in an ensemble was a poor predictor of decoder accuracy, it shows a weak positive correlation in the awake dataset for the primary auditory areas and weak negative correlation in the anesthetized dataset for the secondary auditory areas. The Entropy selectivity which considers both the rate and the temporal patterns of spike trains correlated negatively with decoder performance in the awake dataset and positively with the decoder performance in the primary auditory areas in the anesthetized dataset. The statistical tests are F-tests corrected for the ensemble metrics (see Methods). * $p<0.05$, ** $p<0.01$, *** $p<0.001$. Note that the scale on the x- axis is different in each plot. The scale on the y-axis is different for the awake and anesthetized datasets.

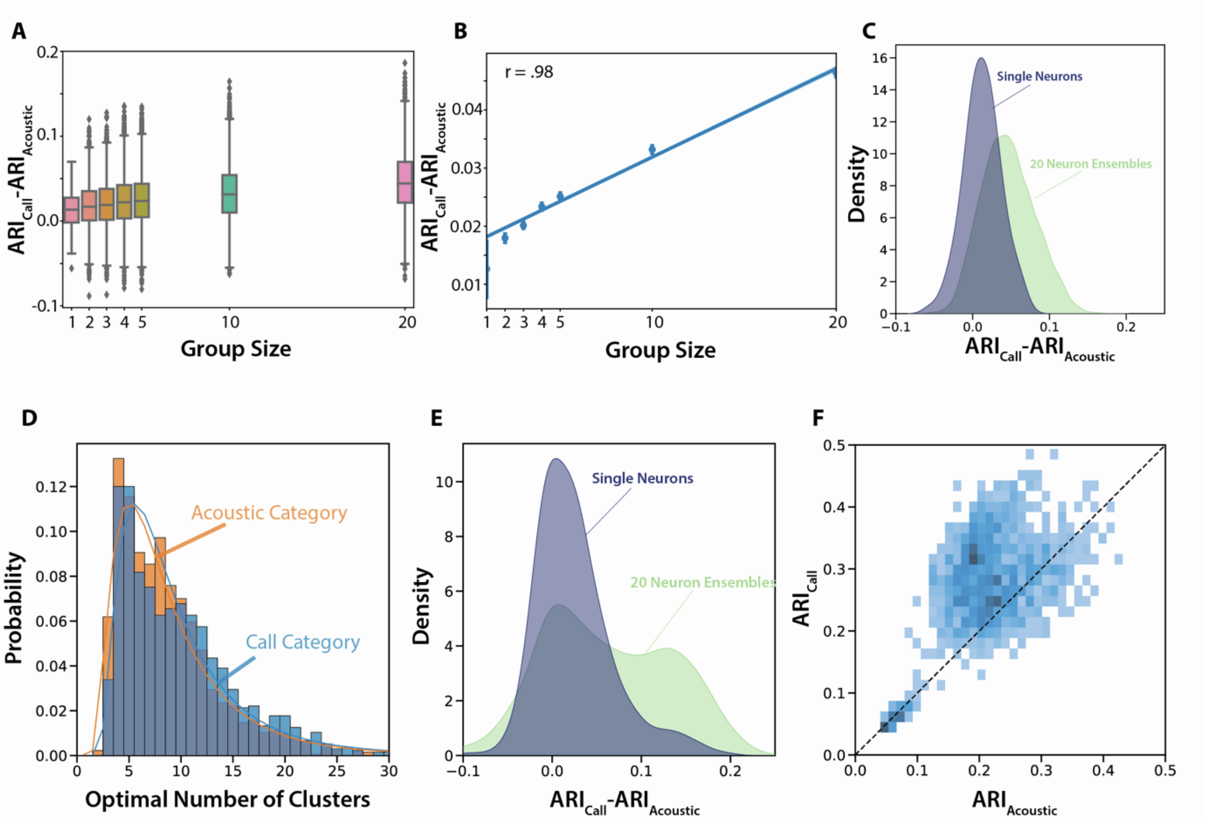

SI Fig 10. A, B, C. Effect of the neuronal ensemble group size on the differences in ARI estimated using the ensemble neural response hierarchical clustering and ethogram-based call-types (ARI_{Call}) or acoustic groups ($ARI_{Acoustic}$) as labels. **Related to Fig. 6.** The differences in ARI is positive and increases with ensemble size, indicating an increased greater match of neural clusters with call-types as compared to acoustic labels. A, B and C are obtained from the awake dataset**. D, E and F** show the distribution of optimal number of neural clusters (D), the distribution of the difference in ARI (E) and the scatter plots of ARI for call-type versus ARI for acoustic labels (F) for the anesthetized dataset. The equivalent of D and F for the awake data are found in panel C of Fig 6 and the equivalent of (E) is found in (C).

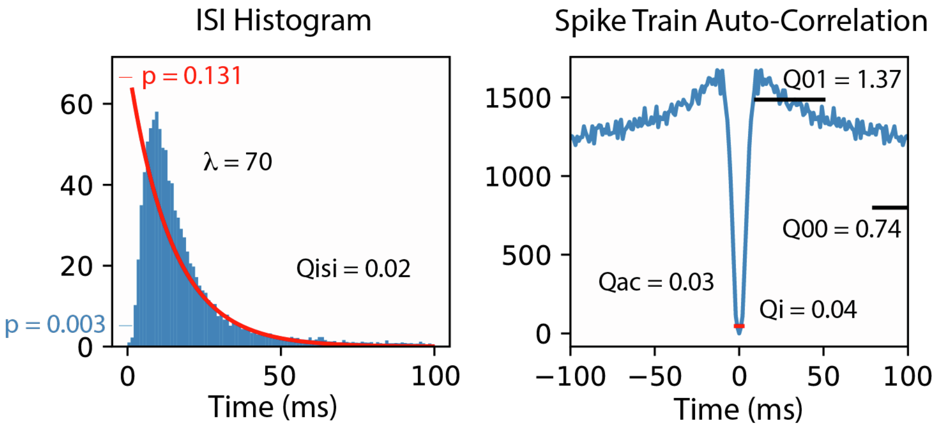

SI Fig 11. Inter-spike interval (ISI) histogram and spike train auto-correlation (AC). Related to STAR Methods. Two quality statistics for the spike sorting procedure were obtained by comparing the measured rate during the refractory period to the expected rate assuming Poisson statistics. The Q_{isi} statistic was obtained by dividing the number of actual ISI obtained in a 2ms window by the expected number from a Poisson distribution. The rate of the Poisson distribution was obtained by fitting an exponential function to the ISI histogram using the 10-100 ms interval. In this example, the Poisson rate is 70 spikes/s (obtained during stimulus presentation). For a recording time of 573 s of stimulus driven activity, we found 77 ISI of less than 2ms (0.3%). The expected number for a Poisson distribution is 3174.65 (13.1%). The Qisi is the ratio of the actual to the expected. Similarly, the Poisson rate can be obtained from the auto-correlation function, which is expected to be flat for a Poisson distribution. Using the same algorithm as in Kilisort2 (github.com/MouseLand/Kilosort/postprocess/ccg.m), two estimates of that rate are obtained: one from 10 to 50 ms time intervals (Q01) and one from the 250 to 500 ms time intervals (Q00). These rate estimates are divided by the overall rate and would be equal to 1 if the firing rate was Poisson. A third estimate of the rate is obtained for the refractory period Qi. We chose t=2.5ms as a delay for the refractory period and estimated the rate from the auto-correlation functions between -2.5 and 2.5 ms. Qi is then divided by the maximum value of Q00 and Q01 to obtain Q_{ac}.
